# High Fat Low Carbohydrate Diet Is Linked to CNS Autoimmunity Protection

**DOI:** 10.1002/advs.202412236

**Published:** 2025-03-27

**Authors:** Duan Ni, Jian Tan, Julen Reyes, Alistair M Senior, Caitlin Andrews, Jemma Taitz, Camille Potier‐Villette, Claire Wishart, Alanna Spiteri, Laura Piccio, Nicholas Jonathan Cole King, Romain Barrès, David Raubenheimer, Stephen James Simpson, Ralph Nanan, Laurence Macia

**Affiliations:** ^1^ Charles Perkins Centre The University of Sydney D17 Charles Perkins Centre Sydney NSW 2006 Australia; ^2^ School of Medical Sciences Faculty of Medicine and Health The University of Sydney Sydney NSW 2006 Australia; ^3^ Sydney Medical School Nepean The University of Sydney Sydney NSW 2747 Australia; ^4^ Nepean Hospital Nepean Blue Mountains Local Health District Sydney NSW 2747 Australia; ^5^ School of Life and Environmental Sciences The University of Sydney Sydney NSW 2006 Australia; ^6^ Sydney Precision Data Science Centre The University of Sydney Sydney NSW 2006 Australia; ^7^ Viral immunopathology Laboratory Infection Immunity and Inflammation Research Theme School of Medical Sciences Faculty of Medicine and Health The University of Sydney Sydney NSW 2006 Australia; ^8^ Brain and Mind Centre The University of Sydney Sydney NSW 2006 Australia; ^9^ The University of Sydney Institute for Infectious Diseases The University of Sydney Sydney NSW 2006 Australia; ^10^ Sydney Nano The University of Sydney Sydney NSW 2006 Australia; ^11^ Novo Nordisk Foundation Center for Basic Metabolic Research University of Copenhagen Copenhagen 2200 Denmark; ^12^ Institut de Pharmacologie Moléculaire et Cellulaire Université Côte d'Azur & Centre National pour la Recherche Scientifique (CNRS) Valbonne 06560 France

**Keywords:** experimental autoimmune encephalomyelitis (EAE), high fat low carbohydrate diet, immunometabolism, macronutrient, multiple sclerosis (MS)

## Abstract

Multiple sclerosis (MS) is an inflammatory and neurodegenerative disease of the central nervous system (CNS) believed to be driven by autoimmune mechanisms. Genetic and environmental factors are implicated in MS development, and among the latter, diets and nutrients are emerging as potential critical contributors. However, a comprehensive understanding of their impacts and the underlying mechanisms involved is lacking. Harnessing state‐of‐the‐art nutritional geometry analytical methods, it is first revealed that globally, increased carbohydrate supply is associated with increased MS disease burden, while fat supply has an opposite effect. Furthermore, in a MS mouse model, experimental autoimmune encephalomyelitis (EAE), it is found that an isocaloric diet high in carbohydrate aggravated EAE, while a diet enriched in fat (HF) is fully protective. This is reflected by reduced neuroinflammation and skewing toward anti‐inflammatory phenotypes. The protective effects from the HF diet are multifaceted. Metabolically, HF increased lipid storage in immune cells, correlating with their increased anti‐inflammatory IL‐10 production. Transcriptionally and epigenetically, HF feeding preprogrammed naïve T cells toward a less activated but more tolerogenic phenotype. It is showcased that manipulating diets is a potentially efficient and cost‐effective approach to prevent and/or ameliorate EAE. This exhibits translational potentials for prevention/intervention of MS and possibly other autoimmune diseases.

## Graphical abstract

Mapping global epidemiological data to macronutrient supply reveals high carbohydrate, in contrast to high fat supply, associates with increased multiple sclerosis (MS) prevalence and incidence. This is confirmed in a mouse MS model, experimental autoimmune encephalomyelitis (EAE), using isocaloric diets: a high carbohydrate diet aggravates, a high protein diet has intermediate effects, and a high fat diet provides complete protection.

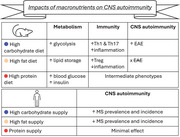

## 1 Introduction

Multiple sclerosis (MS) is an inflammatory, demyelinating and neurodegenerative disease likely to be driven by autoimmune responses. It is characterized by the breakdown of immune tolerance toward myelin, causing damages to the central nervous system (CNS) and resulting in debilitating symptoms including vision loss and paralysis. Despite advances in MS research, its precise aetiology has yet to be defined. MS has been associated with over 200 genes, but its parental disease transmission is low, suggesting a major role for environmental factors in disease development,^[^ ^1^, ^2^ ^]^ including obesity, reduced vitamin D levels and poor diets.^[^ ^3^, ^4^ ^]^

The potential role of diet in MS development was first described in the 1950s, when MS incidence was shown to correlate with high animal fat intake.^[^ ^5^ ^]^ Nevertheless, this study did not consider the role of other macronutrients or correct for calorie intake. The recent rise of MS worldwide has been associated with global “Westernization”, particularly the adoption of a “Western Diet”.^[^ ^6^, ^7^ ^]^ A typical Western diet is high in calories and nutritionally imbalanced, with elevated levels of saturated fats and refined carbohydrates, while poor in dietary fiber. This diet has been shown to exacerbate experimental autoimmune encephalomyelitis (EAE), a mouse model of MS, by promoting inflammatory infiltration into the CNS and inducing a pro‐inflammatory immune profile by favoring pro‐inflammatory T helper (Th) 1 and Th17 polarization while dampening regulatory T cells (Treg) generation.^[^ ^8^, ^9^ ^]^ This is mediated through the activation of p38 mitogen‐activated protein kinase (MAPK) signaling pathway by saturated long‐chain fatty acids.^[^ ^9^ ^]^ In contrast, short‐chain fatty acids (SCFAs), produced by the gut microbiota via the fermentation of complex carbohydrates also known as dietary fiber, have been shown to support immune tolerance through the generation of Treg and regulatory B cells.^[^ ^10^, ^11^ ^]^ Consequently, high‐fiber diets and SCFA supplementation decreased EAE and MS disease severity,^[^ ^9^, ^12^ ^]^ and increased vegetable intake, also enriched in dietary fiber, showed protective effects against pediatric MS.^[^ ^13^ ^]^ Similar trends have been observed with the “Mediterranean diet”, characterized by foods enriched in unsaturated fatty acids and dietary fiber, low in red meats, saturated fatty acids and refined carbohydrates. Its consumption was associated with improved clinical symptoms in MS patients.^[^ ^14^, ^15^ ^]^

Apart from fat and dietary fiber, carbohydrates can also affect immune responses and potentially disease development. We have shown that a high carbohydrate diet boosted B cell development and antibody production.^[^ ^16^ ^]^ Carbohydrates, particularly glucose, are the major source of energy, supporting the activation of immune cells. In this context, inflammation is typically supported by glucose metabolism via glycolysis.^[^ ^17^, ^18^, ^19^, ^20^ ^]^ Diets extremely low in carbohydrates, such as ketogenic diets, have been shown to reduce inflammation and disease severity in EAE^[^ ^21^, ^22^, ^23^, ^24^, ^25^, ^26^ ^]^ and osteoarthritis.^[^ ^27^ ^]^ However, in these studies, the disease pathology was only mildly ameliorated, possibly because the ketogenic diets were not controlled for energy density and nutrient compositions, confounding their analyses. Moreover, most studies applied ketogenic diets as therapeutic interventions, only introducing the diets upon disease model induction or the onsets of disease symptoms. Several important questions remain, like whether a high carbohydrate diet might be a risk factor for EAE/MS, in turn aggravating diseases, and if diets could confer primary preventive effects. Studies with better and proper dietary controls are therefore warranted to address these questions. Finally, the impact of other dietary macronutrient, protein, is also largely unknown, with few reports linking meat serving to Th17 responses in MS.^[^ ^28^ ^]^ However, the presence of saturated fat in meat is also a confounding factor and were not accounted in this study.

Foods and nutrients are the main source of energy for cells and may finetune immune functions by modulating metabolism. While immune cell activation is typically fueled by glycolysis,^[^ ^17^, ^18^, ^19^, ^20^, ^29^ ^]^ an anti‐inflammatory tolerogenic profile is associated with lipid metabolism^[^ ^30^, ^31^, ^32^, ^33^, ^34^ ^]^ and fatty acid oxidation.^[^ ^18^, ^19^, ^29^, ^34^ ^]^ Whether diet may affect immune cell metabolic profiles in MS is unknown.

Here, this study comprehensively interrogated the associations between diet and nutrient environment and MS for the first time. We showed that globally, a nutrient environment with increased carbohydrate supply correlated with higher MS disease burden. A preclinical MS mouse model EAE confirmed a causative effect of diet on EAE severity, with a high carbohydrate diet aggravating EAE by promoting Th1 and Th17 responses and CNS neuroinflammation. Contrarily, an isocaloric diet low in carbohydrate but high in fat protected against EAE by supporting tolerogenic reprogramming.

This work demonstrates that dietary manipulations could be a safe and cost‐effective strategy to prevent and/or control autoimmune diseases.

## 2 Results

### 2.1 Global Carbohydrate Supply Correlated with Increased Multiple Sclerosis Burden

**Figure 1:**
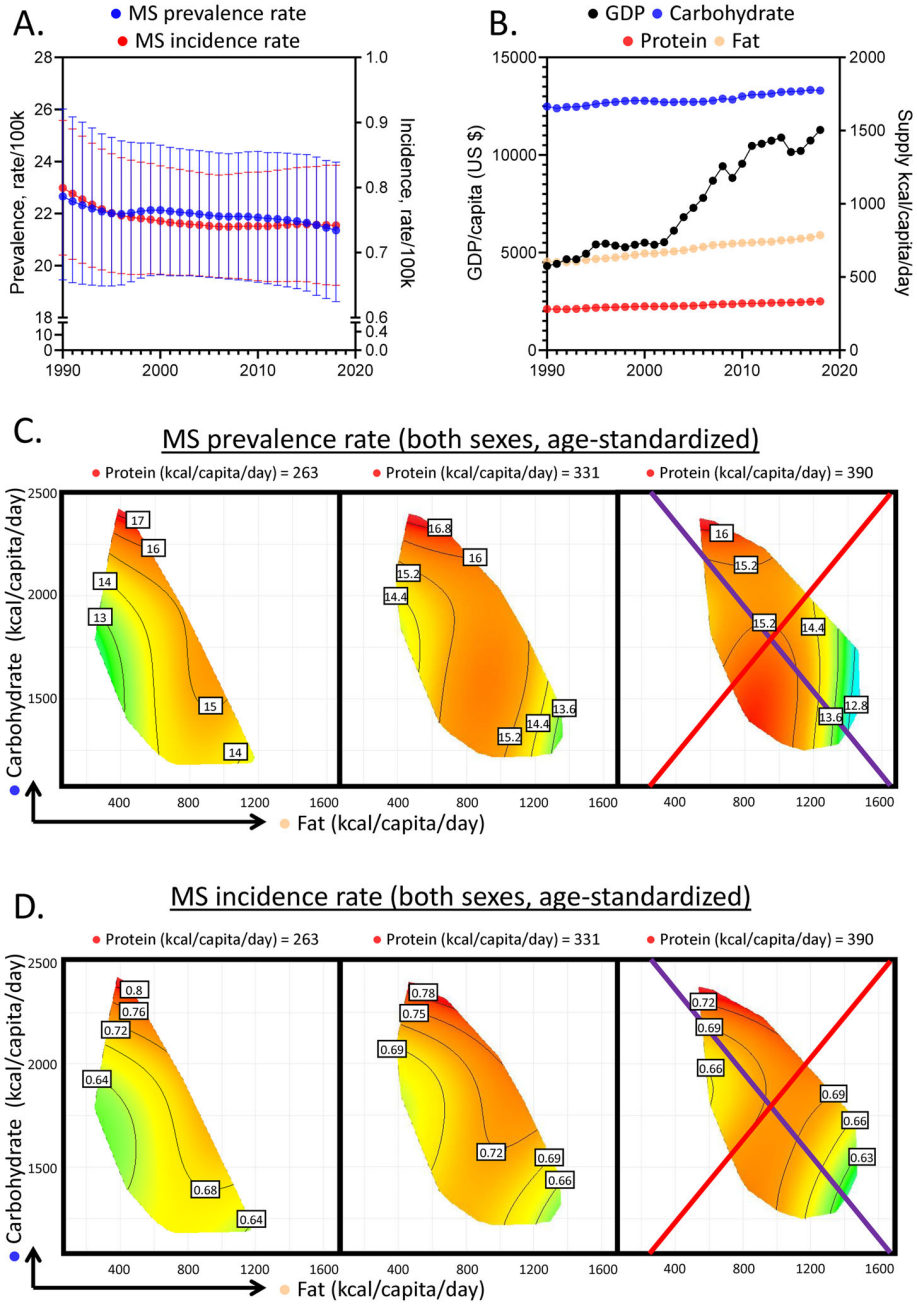
Global association of macronutrient supplies and multiple sclerosis (MS) disease burden. A,B) Age‐standardized MS prevalence (blue) and incidence (red) of both sexes A), global GDP per capita (U.S. dollar, black) and supplies of carbohydrate (blue), fat (light yellow) and protein (red) as functions of year B). C,D) Predicted effects of macronutrient supplies on MS prevalence rate C) and incidence rate D) (See Supporting Information for statistics and interpretation and Figure S1 and Tables S1–S4).

While diet is a key risk factor for MS,^[^ ^35^ ^]^ most studies have only focused on specific dietary patterns or single nutritional factors. These studies neglected potential nutrient‐nutrient interactions and their potential non‐linear effects, which underly many facets of health and diseases,^[^ ^36^, ^37^, ^38^, ^39^ ^]^ as well as broader contexts, such as socioeconomic status. How the nutrient and food environment may affect MS is largely unknown. To address this knowledge gap, we systematically interrogated the association between global macronutrient supplies, a good proxy for dietary environment, and MS disease burden, while adjusting for socioeconomic status and their potential interactions. Macronutrient supplies, disease burden and gross domestic product (GDP) data was collated (Materials and methods). After quality checks, a final dataset containing ∼150 countries from 1990 to 2018 was curated (Figure S1A, Supporting Information). Globally, while both MS prevalence and incidence mildly decreased from 1990 to 2018 (Figure **1**A), macronutrient supplies consistently increased, along with GDP (Figure 1B). These factors are intercorrelated^[^ ^36^, ^38^ ^]^ (Figure S1B–D, Supporting Information), posing significant challenges to disentangle individual association with MS disease burden. Thus, we leveraged cutting‐edge multi‐dimensional nutritional geometric framework (NGF) with generalized additive mixed models (GAMMs) for analyses, which account for inter‐nutrient interactions and their potential non‐linear effects, as well as adjust for other factors like time and GDP.

NGF is a multidimensional analytical method to comprehensively examine individual, additive, and interactive effects of multiple nutritional factors, for example, the 3 macronutrients (protein, carbohydrate and fat), on responses of interest, such as MS prevalence and incidence in this context. NGF represents a powerful tool to disentangle the complex nature of multi‐dimensional nutrient data, conceptualizing nutrition and nutrition effects and interpreting the related outcomes. Its utility has been demonstrated in more than 200 publications involving both animal and human studies, covering numerous areas including metabolism, immunology and longevity.^[^ ^36^, ^37^, ^38^, ^40^, ^41^ ^]^

Here, a series of GAMMs were explored, and a model that considered the interactions between macronutrient supplies and GDP, with an additive effect of time was favored based on Akaike Information Criterion (AIC) calculation (Experimental Section and Supporting Information). This showed that macronutrient supplies together with socioeconomic changes indeed correlated with MS disease burden. GAMM results were visualized in 2D spaces as in Figure 1C,D. Their interpretations are explained further in Supplementary Information and our recent works.^[^ ^36^, ^37^, ^38^ ^]^

Figure 1C presents results for 2018 (Tables S1 and S2, Supporting Information), the most recent year with relatively complete data coverage. Modelled association between MS prevalence and macronutrient supplies were presented as response surfaces within macronutrient supply plots. We focused on fat (*x*‐axis) and carbohydrate (*y*‐axis) supplies while protein was held at 25%, 50% and 75% quantiles of global supply. In these plots, red areas represent higher MS prevalence, reducing toward green. Detailed interpretations are described in Supporting Information and our previous works.^[^ ^36^, ^38^ ^]^

Our modelling revealed that high carbohydrate supply is correlated with higher MS prevalence. High fat, however, was associated with lower prevalence. This is illustrated via the purple isocaloric line. It holds the total macronutrient energy constant but increasing carbohydrate: fat ratio increased MS prevalence (Figure 1C). Protein seemed to confer a moderate effect, as across quantiles of protein supplies, only a mild fluctuation of prevalence was observed. Similar patterns persisted for MS incidence (Figure 1D and Tables S3 and S4, Supporting Information) and held true regardless of sexes (Figure S1E,F, Supporting Information). These findings were minimally confounded by the total energy supply, as increasing total energy while holding carbohydrate: fat ratio constant (red radials) minimally impacted MS disease burden.

These data suggest that globally, carbohydrate supply was associated with higher MS disease burden while fat had an opposite effect, associating with lower MS burden, suggesting the MS protective potential of a low carbohydrate high fat dietary environment.

### 2.2 EAE was Aggravated by High Carbohydrate Diet and Fully Protected by High Fat Diet

The above analyses suggest high carbohydrate supplies as a risk factor for MS, prompting us to investigate the potential causative roles of macronutrients in MS using a murine model, EAE. We fed mice for 6–7 weeks on 3 isocaloric diets with different mixtures of the same macronutrients. They are enriched in carbohydrate (high carbohydrate: HC, Protein (P): Carbohydrate (C): Fat (F) = 5:75:20), fat (high fat: HF, P:C:F = 5:20:75) or protein (high protein: HP, P:C:F = 60:20:20) (Experimental Section and Table S5, Supporting Information). Comparing these 3 diets simultaneously enables us to dissect the roles of different macronutrients and their potential interactions in EAE. For example, comparing HP versus HC and HF could potentially delineate in addition to the amount of protein (60% versus 5% protein), whether combinations with other nutrient components (5% protein + 75% carbohydrate (HC) versus 5% protein + 75% fat (HF)) might play a role and cause any differences.

**Figure 2:**
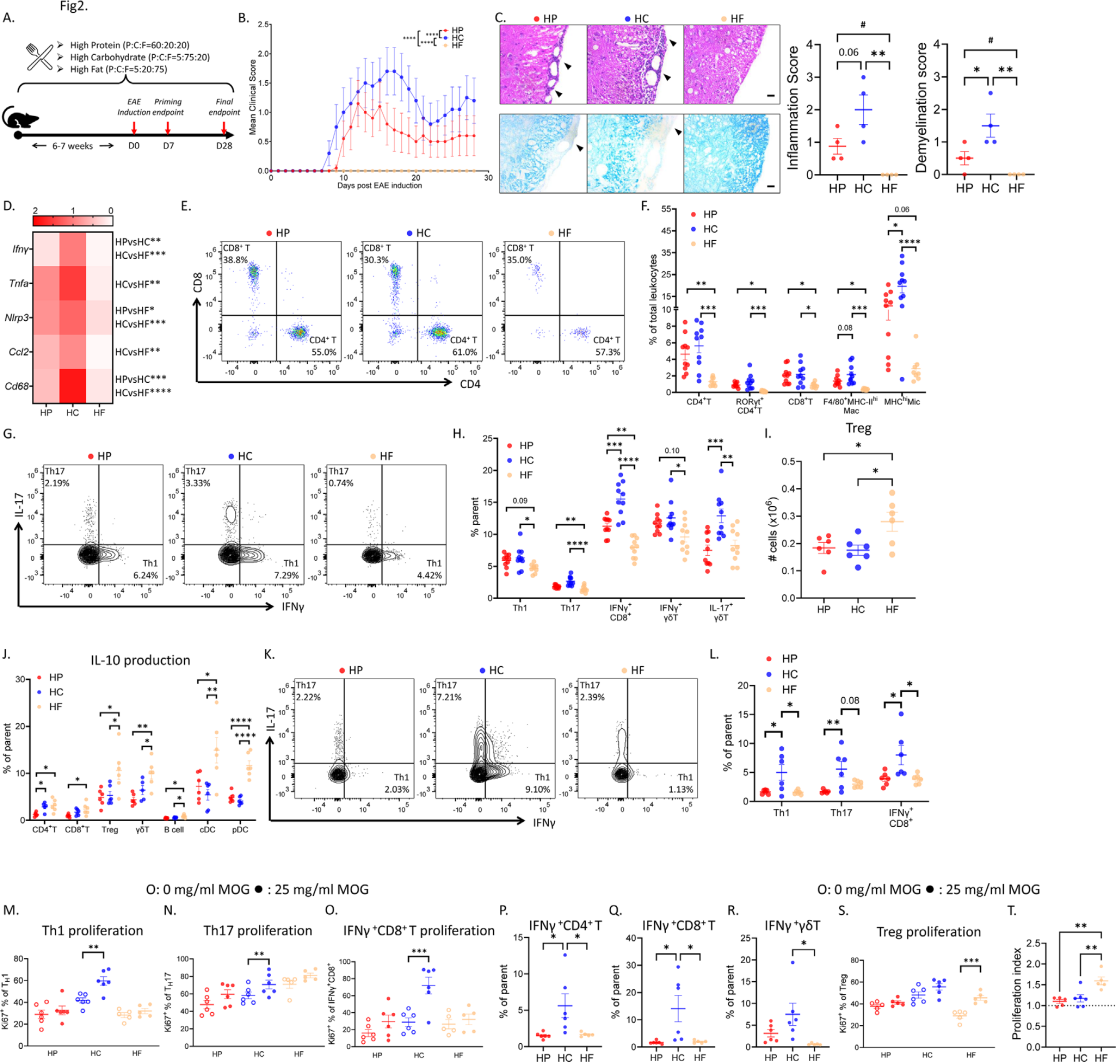
High carbohydrate (HC) feeding aggravated CNS autoimmunity while high fat (HF) was protective. A) Study timeline. Mice were fed on high protein (HP), high carbohydrate (HC) and high fat (HF) diets for 6–7 weeks before experimental autoimmune encephalomyelitis (EAE) induction and were kept on the same diet throughout the EAE clinical course. B) EAE clinical course for HP (red), HC (blue) and HF (light yellow) groups (n = 10/group). C) Histological analysis of neuroinflammation and demyelination of the spinal cord from HP, HC and HF mice isolated on D28 of EAE, paraffin‐sectioned and stained with H&E and Luxol fast blue (LFB) and their corresponding quantification results. Scale bars = 20 µm. D) Heatmap comparisons for the gene expression of *Ifng*, *Tnfa*, *Nlrp3*, *Ccl2*, and *Cd68* in the spinal cord of HP, HC and HF mice isolated on D28 of EAE analyzed by qPCR. E) Representative flow cytometric plots of CNS‐infiltrating T cells of HP, HC and HF mice on D28 of EAE. F) Proportions of infiltrating immune cells and activated microglia (MHC^hi^ Mic) within the CNS of HP, HC and HF mice on D28 of EAE determined by flow cytometry. G) Representative flow cytometric plots of splenic Th1 (IFNγ‐producing CD4^+^ T cells) and Th17 (IL‐17‐producing CD4^+^ T cells) cells of HP, HC and HF mice on D28 of EAE. H) Proportions of pro‐inflammatory cytokine‐producing T cells in the spleens of HP, HC and HF mice were determined by cytometry. I) Total number of Treg in draining lymph nodes (dLNs) of HP, HC and HF mice on D7 of EAE determined by flow cytometry. J) IL‐10 production in immune cells from dLNs of HP, HC and HF mice quantified by flow cytometry. K,L) Representative flow cytometric plots K) and scattering dot plots L) of dLN Th1 and Th17 cells. M–O) Proliferation of dLN Th1 M), Th17 N), and IFNγ‐producing CD8^+^ T cells O) of HP, HC and HF mice, with (filled dots) and without (hollow dots) stimulation, quantified by Ki67 staining. P–R) IFNγ production in dLN CD4^+^ P), CD8^+^ Q) and γδT cells R) of HP, HC and HF mice upon MOG antigen stimulation. S,T) Proliferation of dLN Treg of HP, HC and HF mice, with (filled dots) and without (hollow dots) stimulation S) and their corresponding proliferation indices T), quantified by Ki67 staining, with the dashed line denoting proliferation index of 1. N = 6‐10, data are represented as mean ± S.E.M., with *p < 0.05, **p < 0.01, ***p < 0.001, ****p < 0.0001, by one‐way ANOVA for most analyses; # *p* < 0.05 by unpaired t‐test; two‐way ANOVA for the clinical curve analysis, and paired t‐test for the proliferation assay analysis. (See also Figures S2–S6, Supporting Information).

These diets constitute part of an array of 10 diets (Table S5, Supporting Information) that extensively sample the dietary macronutrient compositions across the protein‐carbohydrate‐fat nutrient space as described previously.^[^ ^16^, ^39^, ^42^ ^]^ Their energy density was held constant by diluting foods with non‐digestible cellulose, allowing ad libitum feeding but restricting total energy intake, a common practice demonstrated in numerous previous studies.^[^ ^16^, ^39^, ^42^, ^43^, ^44^, ^45^, ^46^ ^]^ Of note, the HC diet is close to the composition of AIN‐93G diet (P:C:F = 20:64:16), formulated for animal growth, but with smaller proportion of protein. The HF diet did not reach the threshold for carbohydrate content defining ketogenic diets (<10% of energy),^[^ ^47^ ^]^ and was thus not a ketogenic diet. After 6–7 weeks on diets, EAE was induced as previously described,^[^ ^48^, ^49^ ^]^ while the mice remained on the same diets throughout the experiment (Figure **2**A). Clinical symptoms were monitored daily for 4 weeks as reported.^[^ ^48^ ^]^

Consistent with previous reports using this NGF isocaloric paradigm,^[^ ^42^ ^]^ after 6–7 weeks feeding, despite similar fat mass, mice fed on HF were the leanest while the HP were the heaviest (Figure S2A, Supporting Information), coinciding with highest fasting blood glucose and insulin and their products (similar to homeostatic model‐assessment of insulin resistance, HOMA‐IR, in human) in HP (Figure S2B–D, Supporting Information). During the time course of EAE, bodyweights of mice from HP and HC groups considerably fluctuated, reaching the lowest ∼D13‐14 post EAE induction, coinciding with the peak of disease, while HF exhibited variations to a less extent (Figure S2A, Supporting Information). This was not confounded by food intake, which was comparable across the groups (Figure S2E, Supporting Information). Overall, HC mice developed earlier onset EAE and had higher disease incidence and more severe clinical symptoms, reflected by their higher maximum and cumulative clinical scores, compared to other groups (Figure 2B, Table S6, Supporting Information). In contrast, HP‐fed mice developed intermediate disease severity, and strikingly, HF‐fed mice were fully protected, with zero incidence and clinical score. Notably, mice fed on AIN93G diet, the control diet for mouse growth,^[^ ^50^ ^]^ had similar disease severity to HC, consistent with both diets being high in carbohydrates (Figure S2F, Supporting Information).

Aligned with the disease severity, CNS histopathology from HC‐fed mice displayed the highest inflammatory cell infiltration in the CNS, as well as the most severe demyelination, while HF‐fed mice had little infiltration and no evidence of demyelination (Figure 2C). HP‐fed mice displayed intermediate symptoms (Figure 2C).

Together, these results show that HC diet aggravated EAE, while HF was fully protective. HP diet, low in carbohydrate, but containing the recommended amount of fat (20%) led to an intermediate phenotype. This illustrates that lowering dietary carbohydrate decreases EAE severity, and substituting carbohydrate with fat (HF) instead of protein (HP) is more effective, resulting in full protection against EAE, which highlights the role of dietary macronutrients and their interactions in CNS autoimmunity.

### 2.3 EAE‐Associated Inflammation was Exacerbated by High Carbohydrate Diet and Ameliorated by High Fat Diet

Immune cell infiltration in the CNS during EAE triggers neuroinflammation and tissue damages. Aligned with the aforementioned pathologies, CNS from HC‐fed mice had the highest inflammatory marker gene expression like interferon‐γ (*Ifng)*,^[^ ^51^, ^52^ ^]^ tumour necrosis‐α (*Tnfa)*,^[^ ^53^, ^54^ ^]^ inflammasome *Nlrp3*,^[^ ^55^, ^56^ ^]^ monocyte‐recruiting chemokine *Ccl2* ^[^ ^57^, ^58^, ^59^ ^]^ and macrophage marker (*Cd68)* ^[^ ^60^, ^61^ ^]^ (Figure 2D), all critical mediators of EAE immunopathology. HF‐fed mice exhibited the lowest gene expression, confirming their reduced neuroinflammation, while HP was intermediate. Flow cytometric analyses of CNS infiltrates found the highest proportions and numbers of infiltrating CD4^+^, CD8^+^ and RORγt^+^CD4^+^ T cells (Th17 subset) and inflammatory macrophages (F4/80^+^MHC^hi^) in HC‐fed mice. They also had the highest frequency of activated MHC^hi^ microglia. All these parameters were lowest in HF‐fed mice (Figure 2E,F; Figure S3, Supporting Information). Interestingly, while T cell influx in HP‐fed mice was similar to HC, they had lower inflammatory macrophages and activated microglia (Figure 2F).

Significantly, higher proportions of splenic CD4^+^, CD8^+^ and γδ T cells from HP‐ and HC‐fed mice secreted IFNγ than HF‐fed mice, while HP CD8^+^ T cell IFNγ production was lower than the HC group (Figure 2G,H; Figure S4, Supporting Information). There were also more Th17 cells from HP and HC groups, while HC had the highest IL‐17‐producing γδT cells.

Altogether, these results show exacerbated neuroinflammation and peripheral T cell inflammatory cytokine responses in HC‐fed mice. HF group displayed markedly reduced neuroinflammation, CNS tissue damage, and T cell inflammatory cytokine production, while HP‐fed mice were intermediate for most phenotypes.

### 2.4 High Fat Diet Induced Tolerance during the EAE Induction Phase of T Cell Priming

During EAE, T cells are primed in draining lymph nodes (dLNs) and then recruited to the CNS. There were more T cells (Figure S5, Supporting Information), particularly Treg (Figure 2I), in the dLNs of HF‐fed mice and higher proportions of these T cells secreted anti‐inflammatory cytokine IL‐10 (Figure 2J), suggesting a tolerogenic effect of HF. Highest IL‐10 production was also found for HF B cells and antigen presenting cells including conventional DCs (cDCs) and plasmacytoid DCs (pDCs) (Figure 2J). In contrast, HC had a pro‐inflammatory effect, with significantly more IFNγ and IL‐17 secretion (Figure 2K,L).

T cell priming process was next examined by restimulating dLN lymphocytes *ex vivo* with MOG_35‐55_, the antigen triggering EAE, for 3 days. All groups showed comparable antigen‐specific T cell proliferation, as indicated by the similarly increased proportions of Ki67‐expressing cells and comparable proliferation indices greater than 1 (Figure S6, Supporting Information), suggesting that the priming phase was not impaired among dietary groups. Notably, the proportions of proliferating Th1, Th17 and IFNγ‐producing CD8^+^ T cells were significantly higher only in HC (Figure 2M–O). HC also exhibited the highest proportion of T cell IFNγ production with antigen re‐stimulation (Figure 2P–R). On the other hand, significantly higher proportions of HF Treg showed antigen‐specific proliferation, exhibiting a higher proliferation index (Figure 2S,T).

Collectively, these data demonstrated that HC feeding biased the immune response toward Th1 and Th17, while HF favored a Treg response during EAE priming.

### 2.5 High Fat Diet Shifted Immunometabolism to Support Tolerance in EAE

**Figure 3:**
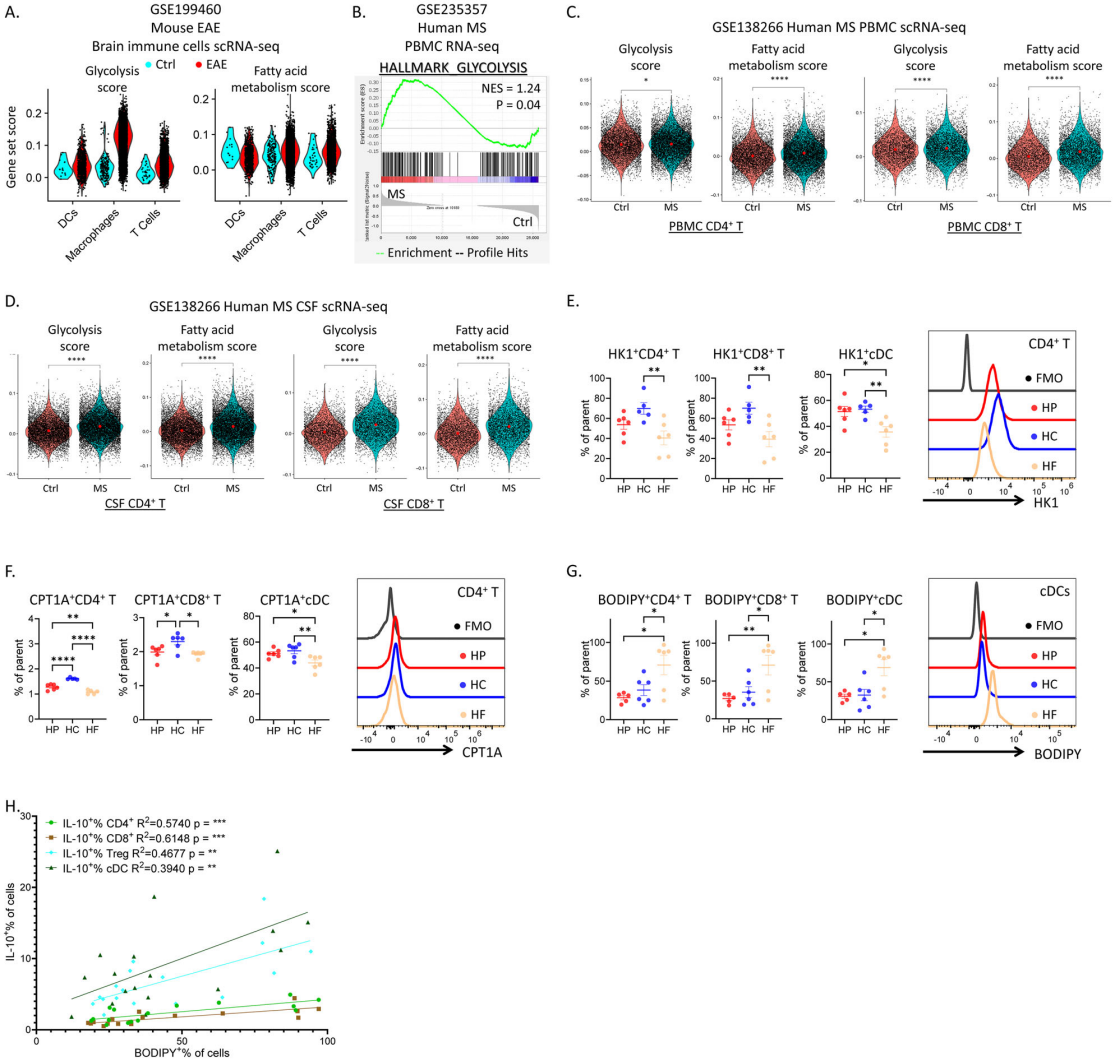
High fat feeding modulated immunometabolism to induce tolerance during CNS autoimmunity. A) Violin plots of the gene set scores for glycolysis and fatty acid metabolism for CNS‐infiltrating immune cells in Ctrl (cyan) and EAE (red) mice analyzed by scRNA‐seq. B) Gene set enrichment analysis (GSEA) showing the enrichment of glycolysis pathway in the peripheral blood mononuclear cells (PBMCs) from MS patients versus Ctrl. C,D) Violin plots of the gene set scores for glycolysis and fatty acid metabolism of T cells in PBMCs (C) and cerebrospinal fluid (D, CSF) from Ctrl (pink) and MS patients (blue) analyzed by scRNA‐seq. E–G) Levels of HK1 (E), CPT1A (F) and lipid‐staining BODIPY 493/503 (G) in dLN T cells and conventional dendritic cells (cDC) of HP, HC and HF mice on D7 of EAE were quantified by flow cytometry. H) IL‐10 production (IL‐10^+^% of cells) in different immune cell subsets (CD4^+^ T cells (green), CD8^+^ T cells (brown), Treg (cyan) and cDC (dark green)) was significantly correlated with their corresponding lipid content (BODIPY^+^% of cells), as quantified by BODIPY 493/503 staining. (See also Figures S5, S7–S9, Supporting Information). N = 6‐10, data are represented as mean ± S.E.M., with *p < 0.05, **p < 0.01, ***p < 0.001, ****p < 0.0001, by one‐way ANOVA.

Diet supplies substrates fueling metabolic pathways that support immune cell functions. Immunometabolic disruption has been reported in autoimmunity, particularly in EAE.^[^ ^18^, ^29^ ^]^ We investigated the immunometabolic profiles of CNS‐infiltrating cells in EAE by single‐cell RNA sequencing (scRNA‐seq) from a published dataset.^[^ ^62^ ^]^ We calculated the Hallmark gene set scores for glycolysis and fatty acid metabolism, two key pathways implicated in immune cell functions, particularly in T cells. We found that during EAE, T cells, DCs and macrophages exhibited significantly higher glycolysis scores (Figure **3**A), presumably linked to their enhanced inflammatory phenotypes (Figure S7A, Supporting Information). Surprisingly, fatty acid metabolism scores were also higher (Figure 3A), potentially reflecting the higher energy demand required for immune activation and inflammation. Similarly, gene set enrichment analysis (GSEA) of RNA‐seq data of total peripheral blood mononuclear cells (PBMCs) from MS patients showed an increased glycolysis signal (Figure 3B). This was further supported by scRNA‐seq analysis of blood and cerebrospinal fluid T cells. As in EAE, T cells from MS patients had significantly higher glycolysis and fatty acid metabolism scores and inflammatory signals than healthy individuals (Figure 3C,D; Figure S7B, Supporting Information). Altogether, we found that immunometabolism, particularly glycolysis, is enhanced during CNS autoimmunity.

To investigate how diets impact on immunometabolism, enzymes and markers involved in critical metabolic pathways were analyzed using MetFlow, a flow cytometry‐based method achieving single cell‐level metabolic profiling with comparable robust performances to conventional approach like Seahorse experiments.^[^ ^17^ ^]^ Hexokinase 1 (HK1) was validated as a readout of glycolysis,^[^ ^17^ ^]^ a critical mediator of inflammatory responses, specifically those driven by T cells.^[^ ^17^, ^18^, ^19^, ^20^ ^]^ In dLNs, HK1 was upregulated in HC‐derived T cells, as well as in cDCs, compared to HF (Figure 3E). This demonstrated that HC promoted glycolysis, which likely fuelled their enhanced immune activation and disease severity. Consistent with their reduced inflammation, HF displayed the lowest proportions of HK1^+^ cells. In HP, although HK1^+^ T cells were lower than HC, this was not significant. However, proportions of HP cDCs expressing HK1 were comparable to HC, significantly higher than HF.

Carnitine palmitoyltransferase 1A (CPT1A) is involved in fatty acid oxidation, associated both with immune activation^[^ ^32^, ^33^ ^]^ and tolerance.^[^ ^63^ ^]^ Higher CPT1A levels in T cells and cDCs were found in HC group with more severe symptoms (Figure 3F), consistent with enhanced fatty acid metabolism during EAE/MS revealed by scRNA‐seq.

Cellular fat storage, generally associated with tolerogenic phenotypes,^[^ ^19^, ^20^, ^30^, ^31^, ^33^ ^]^ was quantified with BODIPY 493/503 staining. Significantly higher proportions of HF T cells and cDCs were BODIPY^+^ (Figure 3G), illustrating increased fat accumulation accompanying HF feeding. Such effects were evidently systemic, as circulating HF T cells had higher fat storage throughout the course of EAE (Figure S8, Supporting Information). Importantly, lipid contents in T cells and cDCs were significantly correlated with their IL‐10 production, but not other cytokines (Figure 3H; Figure S9, Supporting Information), suggesting that fat accumulation in immune cells may contribute to IL‐10 production and thus immune tolerance.

In summary, HC diet enhanced glycolysis and fatty acid oxidation in T cells and DCs while HF increased fat storage in most immune cells and correlated with higher IL‐10 production, suggesting a tolerogenic impact.

### 2.6 High Fat Diet Pre‐Conditioned Immune Cells Toward Tolerogenic Phenotypes

As HF induced tolerance in EAE, we next investigated whether HF diet could pre‐condition immune cells to tolerogenic phenotypes under basal conditions. Splenic DCs from different dietary groups were examined for their activation status based on their MHC‐II expression. HF‐derived DCs had the lowest levels of MHC‐II, implying their reduced activation (Figure S10A, Supporting Information). This pattern persisted during EAE (Figure S10B, Supporting Information), possibly accounting for the reduced inflammatory responses in HF group.

**Figure 4:**
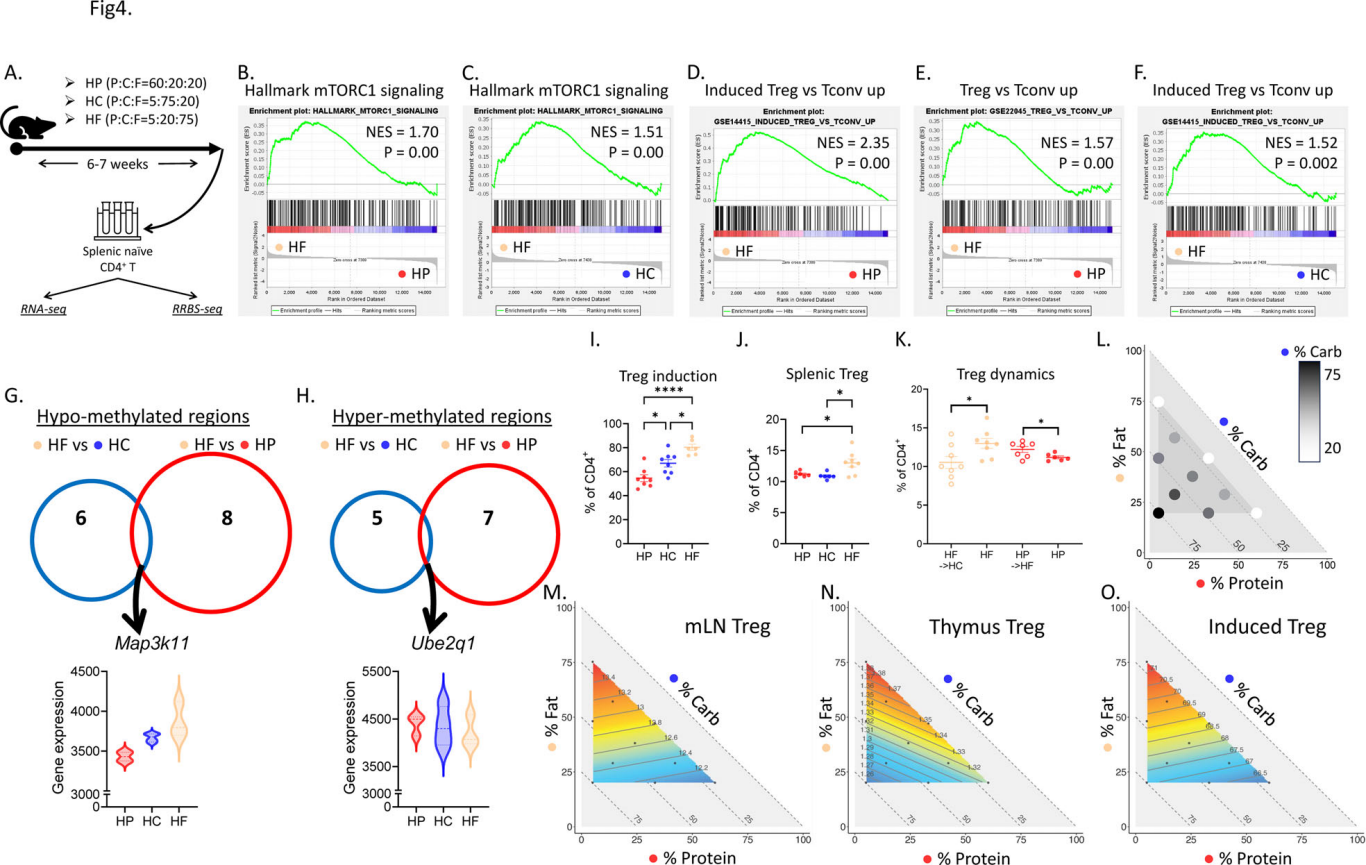
High fat feeding preconditioned T cell toward Treg differentiation. A) Experimental design. Mice were fed on HP, HC and HF diets for 6–7 weeks before sorting splenic naïve CD4^+^ T cells for RNA‐sequencing (RNA‐seq) and reduced‐representation bisulfite sequencing (RRBS‐seq). B,C) GSEA showing the enrichment of mTORC1 signaling pathway comparing HF naïve T cells with HP (B) and HC (C). D–F) GSEA showing the enrichment of signals upregulated in Treg versus conventional T cells comparing HF naïve T cells with HP (D,E) and HC (F). G,H) Venn diagrams showing the hypo‐methylated (G) and hyper‐methylated (H) regions comparing HF naïve T cells with HP (red) and HC (blue) and their overlapping genes *Map3k11* (hypo‐methylated) and *Ube2q1* (hyper‐methylated), and the violin plots for the expression of *Map3k11* and *Ube2q1* analyzed by RNA‐seq. I) Proportions of Treg differentiated from splenic naïve CD4^+^ T cell from HP (red), HC (blue) and HF (light yellow) mice quantified by flow cytometry. J) Proportion of splenic Treg from HP, HC and HF mice. K) Proportion of splenic Treg from mice fed on HF diet and mice first fed on HF diet and then switched to HC diet (HF→HC), and mice fed on HP diet and mice fed on HP diet then switched to HF diet (HP→HF). L) Visualization of the compositions of diets used in this study. Each circle represents one diet and their relative locations on the *x*, *y* and hypotenuse axes denote the proportion of protein, fat and carbohydrate (carb). The proportions of carbohydrate are also reflected by the color range. M–O) Contributions of macronutrient compositions to Treg proportions in mesenteric lymph node (M, mLN) and thymus (N) and to in vitro Treg differentiation experiment (O), were modelled by mixture modelling and mapped on right‐angled mixture triangles, consisting of protein (*x*‐axis), fat (*y*‐axis) and carbohydrate (hypotenuse). (See Supporting Information for statistics and interpretation and Table S5–S8). N = 6‐8, data are represented as mean ± S.E.M., with *p < 0.05, **p < 0.01, ***p < 0.001, ****p < 0.0001, by one‐way ANOVA for most analyses and unpaired t‐test for the Treg dynamics analysis.

In addition to DCs, T cells play a central role in autoimmunity like EAE. We thus tried to inspect their potential differential pre‐programming under naïve conditions. We first analyzed the properties of splenic naïve CD4^+^ T cells from HP‐, HC‐, and HF‐fed mice by RNA‐seq (Figure **4**A). GSEA, which comprehensively profiled changes within the whole transcriptome, revealed HF naïve T cells exhibited enhanced mTORC1 signal (Figure 4B,C), which is critical for Treg generation and function.^[^ ^64^, ^65^ ^]^ Importantly, they were also transcriptionally more primed to differentiate toward a Treg profile, with enrichment in gene sets upregulated in Treg compared to conventional T cells (Figure 4D–F).

Epigenetic changes are well known to influence T cell differentiation and activation.^[^ ^66^, ^67^, ^68^ ^]^ The epigenetic profile of naïve T cells from the three dietary groups was analyzed through reduced representation bisulfite sequencing (RRBS‐seq) for genome‐wide methylation profiles. Compared to HP and HC, HF naïve CD4^+^ T cells were consistently hypo‐methylated in a CpG island in the *Map3k11* promoter region, and hyper‐methylated in the promoter region of *Ube2q1* (Figure 4G,H, and Table S7, Supporting Information). Hypo‐methylation of promoter regions is generally associated with enhanced gene expression. Accordingly, we confirmed by RNA‐seq the increased expression of *Map3k11*, as well as its downstream target gene *Ppia* ^[^ ^69^ ^]^ in HF group (Figure 4G; Figure S11, Supporting Information). As downregulation of Map3k11 and Ppia was linked to T cell activation and pro‐inflammatory functions,^[^ ^69^ ^]^ their upregulation may explain the anti‐inflammatory role of HF.

To confirm these results, we induced the differentiation of naive T cells isolated from HP‐, HC‐ and HF‐fed mice toward Treg e*x vivo*. We found that the Treg differentiation was significantly higher in the HF group (Figure. 4I), consistent with its tolerogenic phenotype. HF feeding for 6 weeks also prominently increased splenic Treg (Figure 4J). This effect was dynamic, as Treg was decreased when switching HF to HC feeding for 6 weeks (Figure 4K).

Finally, to comprehensively confirm that fat was the main macronutrient driving Treg generation, we subjected mice to one of the ten isocaloric diets (Table S5, Supporting Information) spanning the nutritional space in Figure 4L for at least 6 weeks. These diets covered different macronutrient compositions from 5%–60% protein, 20%–75% carbohydrate and 20%–75% fat. The impacts of dietary macronutrients on Treg proportions were analyzed with NGF mixture models and visualized using a proportion‐based nutritional geometry model as in our previous works.^[^ ^16^, ^42^ ^]^ In brief, the predicted effects of macronutrient compositions on Treg proportions were mapped on right‐angled mixture triangle plots as in Figure 4M–O, where the *x* and *y* axes presented the protein and fat proportions in diets, and the hypotenuse was for carbohydrate. Regions within the modelling surfaces in red indicated higher Treg proportions, while the ones in blue meant lower, with numbers on the isolines denoting the predicted Treg frequencies. Four mixture models^[^ ^70^ ^]^ were fitted with our data and model 1 was chosen based on AIC evaluation (Materials and methods). This indicated that dietary fat concentration was the major driver of Treg frequency. Strikingly, in the mesenteric lymph nodes (mLNs) and thymus, there was a clear increase of Treg proportions with increasing dietary fat contents (Figure 4M,N; Tables S8 and S9, Supporting Information). A similar fat‐driving pattern was also found for the Treg polarization assay results from different dietary groups (Figure 4O, Table S10, Supporting Information).

Collectively, these data show that dietary fat is the main dietary driver of Treg generation by preconditioning naïve T cell toward Treg differentiation.

## 3 Discussion

**Figure 5:**
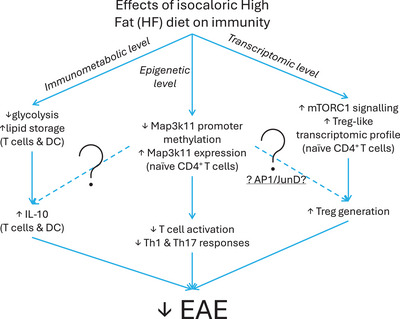
Overview of the potential mechanisms underlying the multifaceted anti‐inflammatory effects of an isocaloric high fat (HF) diet. HF reprogrammed the immunometabolism, reducing the pro‐inflammatory glycolysis while promoting the anti‐inflammatory lipid storage in T cells and dendritic cells (DC), which led to a more tolerogenic phenotype as reflected by higher IL‐10 production. HF also modulated naïve CD4^+^ T cell at epigenetic and transcriptomic levels. HF reduced the promoter methylation of Map3k11 and increased the gene expression, which was linked to dampened T cell activation and reduced Th1 and possibly Th17 responses. Higher Map3k11 expression might associate with increased IL‐10 production and enhanced Treg generation, via activation of AP1/JunD signals, which warranted future investigations. HF also upregulated the mTORC1 signaling in naïve CD4^+^ T cells and skewed them toward a transcriptomic profile more similar to Treg, promoting Treg generation. Together, through these multi‐factorial mechanisms, HF feeding was linked to protection against experimental autoimmune encephalomyelitis (EAE).

In summary, this study reveals for the first‐time the causative roles for macronutrients in preventing CNS autoimmunity. Global ecological analyses revealed a positive correlation between the carbohydrate supply and MS disease burden, while fat supply had the opposite effect. Mechanistically, in a preclinical EAE model, we found that HC aggravated disease severity, while HF was fully protective. Leveraging isocaloric standardized diets and avoiding confounders like diet‐induced obesity and metabolic disorders, we showed that HF primed toward tolerogenic phenotypes by modulating immune cell metabolism, particularly through enhanced lipid storage. HF feeding also preconditioned naive T cell toward Treg differentiation, possibly via transcriptomic and epigenetic modifications. These multifaceted changes translated into EAE protection (Figure **5**), suggesting that dietary manipulation could be a strategy to prevent and/or modulate CNS autoimmunity severity.

Diets and nutrients are known to be implicated in MS pathogenesis, but in‐depth understanding is lacking, and their potential protective prospects are also underappreciated. Recently, there have been attempts to treat established MS with very‐low‐carbohydrate‐high‐fat ketogenic diets, with some therapeutic effects.^[^ ^71^, ^72^ ^]^ However, the potential roles of diets and dietary macronutrients in MS as primary preventions or underlying risk factors remain to date largely unknown.^[^ ^7^, ^35^, ^72^ ^]^ In this regard, our global ecological analyses revealed that high‐fat‐low‐carbohydrate food environments were indeed linked to reduced MS incidence and prevalence, suggesting high carbohydrate dietary patterns as a potential risk factor for MS while high fat dietary patterns as protective.

These findings prompted us to investigate the underlying mechanisms. Our preclinical study unveiled that increasing carbohydrates enhanced immune activation and exacerbated autoimmune neuroinflammation in an isocaloric context. This seems to be mediated via the activation of the glycolysis pathway, known to fuel immune cell activation and previously reported to be involved in various T cell‐mediated inflammation and autoimmunity.^[^ ^17^, ^18^, ^19^, ^20^ ^]^ These effects by HC aligned with our previous findings that HC supported B cell differentiation and functions through glycolysis.^[^ ^16^ ^]^ While murine EAE is largely T cell‐driven, B cells are critical in human MS, suggesting a potential role of carbohydrate in MS development involving multiple immune cell subsets.

Lowering carbohydrate alone, however, seems inadequate to achieve full protection against EAE. Our HP contained the same carbohydrate content as HF, but still led to intermediate EAE pathology. This indicated that substituting carbohydrate with protein is less effective for EAE protection in diets. This might be due to their worst overall metabolic health reflected by their highest fasting blood glucose and insulin, which are known to exacerbate EAE.^[^ ^21^, ^49^, ^73^, ^74^ ^]^ These results also highlight the critical role of the combination of low‐carbohydrate‐high‐fat for complete EAE protection.

Some previous studies have tested very‐low‐carbohydrate‐high‐fat ketogenic diets in EAE.^[^ ^21^, ^22^, ^23^, ^24^, ^25^, ^26^ ^]^ However, they failed to stringently control the energy density and types of macronutrients in diets. Also, in most studies, diets were introduced upon EAE induction or after EAE onset,^[^ ^21^, ^22^, ^25^, ^26^ ^]^ with only two prior to EAE induction.^[^ ^23^, ^24^ ^]^ In two studies interrogating the preventive effects of ketogenic diets, they only started the diets 10–14 days before EAE induction, a relatively short timeframe for dietary interventions. Consequently, these factors might all account for the fact that others generally only found an incomplete amelioration or protection of the EAE symptoms. Here, we controlled carefully our diet design, ensuring similar energy density and macronutrient types across our diets, only varying their ratios. We also initiated our dietary intervention 6–7 weeks prior to EAE induction as a preventive measure. These might together contribute to our observed full protection against EAE by HF. More importantly, harnessing the state‐of‐the‐art nutritional geometry approaches, our analyses focused on more than an individual dietary pattern like ketogenic diets, but on the overall nutritional landscape, shedding unprecedented comprehensive overviews on the effect of macronutrients on EAE. In this context, our HF diet is by definition not a ketogenic diet, which required its carbohydrate composition to be lower than 10% of the total energy content.^[^ ^47^ ^]^ This might provide a novel alternative dietary regimen for clinical MS prevention or treatment, if further validated. On the other hand, HF is based on fat derived from canola oil. Some ketogenic diets in the literature contained saturated fat derived from butter and lard,^[^ ^21^ ^]^ which was previously shown to aggravate EAE.^[^ ^9^ ^]^ More in‐depth studies comparing different diets and their macronutrient components, characterizing both the quantitative and qualitative influences of macronutrients on immunity are needed.

The anti‐inflammatory effects of HF are multifaceted (Figure 5), warranting further investigations to elucidate the detailed contributions from different aspects, such as more in‐depth profiling of the immune‐metabolism crosstalk and the related immune functions. We reported that upon HF feeding, beyond T cells, other immune cell types also produced more IL‐10. This correlated with their higher fat accumulation in cells, which is aligned to previous reports.^[^ ^20^, ^30^ ^]^ This suggests that surpassing a specific threshold of cellular fat storage might induce a tolerogenic profile. This also extended to the blunted activation in HF‐derived DC in our data and is supported by previous reports that lipids and fatty acids are linked to anti‐inflammatory responses.^[^ ^20^, ^30^, ^31^, ^75^, ^76^, ^77^ ^]^ This might be a conserved mechanism, considering its implications in multiple immune cell types. Surprisingly, despite higher fat contents, HF immune cells did not exhibit increased fatty acid oxidation, as there are fewer CPT1A^+^ cells from HF. Although fatty acid oxidation is frequently linked to immune tolerance, its disruption like CPT1A inhibition in mice or mutations in humans led to reduced neural autoimmunity,^[^ ^78^, ^79^ ^]^ similar to our findings with HF. This suggests that other mechanisms, either metabolic or non‐metabolic, may be involved. Among the “non‐metabolic” effects of HF diet, epigenetic changes may play a role through the decreased methylation of *Map3k11* promoter, leading to its increased gene expression and to the upregulation of its downstream target *Ppia*.^[^ ^69^ ^]^ Since *Map3k11* and *Ppia* inhibition was previously shown to induce T cell activation and inflammatory cytokine production,^[^ ^69^ ^]^ the higher expression of *Map3k11* and *Ppia* may dampen T cell activation. This might explain the blunted T cell activation under HF feeding condition in EAE. Furthermore, Map3k11 can activate downstream signaling involving AP1 and JunD, which might regulate the expression of an array of Treg effector molecules including Foxp3.^[^ ^80^, ^81^ ^]^ This might also underly the link between increased Map3k11 levels and bias toward Treg differentiation in HF, although further validation is needed. On the other hand, Treg generation and Foxp3 expression are known to be modulated by DNA methylation,^[^ ^82^, ^83^ ^]^ and the role of Map3k11 in this requires further investigations. How HF influences DNA methylation is unknown. Potential explanations include the reduction in methionine and thus its derivative S‐adenosylmethionine, the methyl donor in methylation reactions, in diets like HF low in protein;^[^ ^84^ ^]^ or the increase in adenosine, further suppressing DNA methyltransferases,^[^ ^85^, ^86^ ^]^ as reported in studies using ketogenic diets, which are also high in fat. Finally, diets enriched in fat and cellular lipids could induce other epigenetic modifications such as acetylation, known to modulate Treg differentiation.^[^ ^87^, ^88^, ^89^, ^90^, ^91^ ^]^

Importantly, HF feeding induced a transcriptomic profile in naive CD4^+^ T cells similar to Treg's, which might bias their differentiation toward Treg. This intrinsic preconditioning of naive T cells could explain the increased Treg generation across organs driven by dietary fat, as shown by our mixture modelling analyses. Moreover, it would be important to determine whether these changes are long‐lasting, particularly for the epigenetic imprinting, although our data suggested that the alterations in Treg by dietary manipulation might be dynamic.

Finally, an HP diet resulted in an intermediate EAE severity, worse than HF but better than HC, suggesting a distinct role of protein in EAE. Despite not being the primary energy source for T cells, amino acids still play critical parts in their activation and function,^[^ ^92^ ^]^ possibly explaining the enhanced pathology by HP compared to HF feeding conditions. Other indirect effects, beyond the scope of this study, like changes in gut microbiota,^[^ ^42^ ^]^ as we previously documented, may contribute to the differential effects of macronutrients on EAE severity.

In summary, our results suggest that dietary manipulation is an effective method to modulate immune responses and prevent/control EAE. Although supported by our population‐level ecological analyses, the translation potentials of our discoveries need to be confirmed. If validated, these findings suggest that diet could be used as a safe and cost‐effective immunomodulatory MS prevention and/or intervention and for potentially other autoimmune diseases. It might also emerge as a promising prevention strategy in populations at risk or as an adjuvant for current treatments.

## 4 Experimental Section

### Mice and Diets

Female C57BL/6 mice were purchased from Australian Animal Resources Centre and were housed at the Charles Perkins Centre, the University of Sydney, under specific‐pathogen‐free conditions, with a 12 h light/dark cycle (6pm‐6am), at 22 °C, 50% humidity. All animal experiments were approved by the University of Sydney Animal Ethics Committee (Protocol ID 1737).

Mice were fed ad libitum on diets listed in Table S5 (Supporting Information). These ten diets were designed isocalorically (14.5 MJ kg^−1^) based on the AIN‐93G diet, the control diet for animal growth, only modifying their macronutrient (protein, carbohydrate and fat) compositions. They covered a macronutrient range of 5%–60% protein, 20%–75% carbohydrate and 20%–75% fat, chosen based on nutritional geometry analyses to comprehensively sample dietary macronutrient mixture, as reported in previous publications from the team.^[^ ^16^, ^39^, ^42^ ^]^ The high protein (HP, P60/C20/F20), high carbohydrate (HC, P5/C75/F20), and high fat (HF, P5/C20/F75) diets reflect the extremes (apices) diets among these ten diets. All diets, including the AIN‐93G diet, were purchased from Specialty Feeds, Gleen Forest, Australia.

### Flow Cytometric Analysis

For spleens, thymus, and lymph nodes, organs were disrupted mechanically to prepare a single cell suspension, which were then filtered through 100 µm cell strainers and underwent red blood cell (RBC) lysis using 1x RBC lysis buffer (BioLegend). The resulting samples were then resuspended in fluorescence‐activated cell sorting (FACS) buffer (2% fetal bovine serum (FBS) in phosphate‐buffered saline (PBS) containing 1 mM disodium ethylenediaminetetraacetate (EDTA)) until further processing.

For central nervous system, brains and spinal cords were first mechanically disrupted and filtered. Next, the resulting suspension was centrifuged for 10 min at 300 x g at 4 °C to pellet the cells, which further went through 30%/37%/70% Percoll gradient centrifugation for 20 min at 1200 x g at 4 °C without the brake for isolation and enrichment. After that, cells were washed and resuspended in FACS buffer until further processing.

For cytokine quantification, cells were cultured in complete RPMI culture media supplemented with phorbol 12‐myristate 13‐acetate (PMA), ionomycin, and brefeldin A for 4 h in incubators with 5% CO_2_ at 37 °C, followed by intracellular staining.

For intracellular staining, cells were first permeabilized and fixed using the Foxp3/transcription factor staining buffer kit (eBioscience) following the manufacturer's protocol and then stained with the corresponding intracellular antibodies.

Antibodies used in this study were listed in Table S11 (supporting Information). Data was recorded on a 5‐laser Aurora spectral cytometer (Cytek Biosciences, USA) or with a BD LSR‐II analyzer (Becton Dickinson) using the FACSDiva software. Data was analyzed with FlowJo v10.9.0. (Treestar Inc. Ashland) based on the gating strategies described in Figures S3–S6, S8 (Supporting Information).

### MetFlow Staining

MetFlow markers were obtained from previous published study.^[^ ^17^ ^]^ In brief, HK1 (hexokinase 1) was chosen as a marker for glycolysis, and CPT1A (carnitine palmitoyl‐transferase 1A) was selected as marker for fatty acid oxidation. Anti‐HK1 (ab150423) and anti‐CPT1A (ab128568) primary antibodies were purchased from Abcam and then conjugated with PE/Cy7 and AF700 fluorophores using conjugation kits from Abcam (ab102903 and ab269824) respectively.

MetFlow staining was done in parallel with conventional cytometric intracellular staining. In brief, cells were permeabilized and fixed with the Foxp3/transcription factor staining buffer kit (eBioscience) following the manufacturer's protocol and then stained with the corresponding antibodies.

For lipid staining, cells were stained with BODIPY 493/503 (D3922, Invitrogen) following the manufacturer's protocol.

### Induction and Evaluation of Experimental Autoimmune Encephalomyelitis (EAE)

For EAE experiment, each mouse was injected with 50 µg myeline oligodendrocyte glycoprotein (MOG_35‐55_: MEV GWY RSP FSRVVH LYR NGK; GenScript) emulsified in incomplete Freund's adjuvant (Chondrex, Inc.) and 50 µg desiccated *Mycobacterium tuberculosis* (strain H37RA). On the same day of immunization and 2 days after, mice received 300 ng mouse^−1^ pertussis toxin (List Biological Laboratories) via intravenous injection.

After EAE induction, clinical symptoms of the mice were monitored following the criteria described in Table S12 (Supporting Information). Clinical courses were evaluated for at least 4 weeks after EAE induction by 2 independent researchers in a blinded manner and experiments were repeated twice.

### Ex Vivo Proliferation Assay for EAE Experiments

*Ex vivo* T cell proliferation assay for antigen‐restimulation were carried out on D7 post EAE induction. Draining lymph nodes (dLNs, axillary, cervical and inguinal lymph nodes) were mechanically disrupted to prepare single cell suspension. Next, cells were cultured in 200 µL complete RPMI media with or without 25 µg ml^−1^ MOG at 2 × 10^5^ cells per well in round‐bottom 96‐well plates for 72 h. After culture, cells were washed with PBS and analyzed by flow cytometry for intracellular Ki67 staining as readout of proliferation. Proliferation index was calculated as described in the previous works.^[^ ^73^ ^]^

### Histological Analyses

Upon sacrifice, mice were perfused with cold 4% paraformaldehyde and then the CNS tissues were dissected and fixed in 4% paraformaldehyde for 24 h. Tissues were next washed with PBS and decalcified in 0.5 M EDTA buffer for 7 days. The decalcifying buffer was changed every 2 days and samples were rocked at room temperature. After decalcification, samples were washed with PBS for 1 h and then went through sequential dehydration with 30% and 50% ethanol for 30 min before storage in 70% ethanol until further processing.

Histology sections were prepared and stained following standard protocols.^[^ ^48^ ^]^ Tissues were first embedded in paraffin and cut in 4 µm sections, followed by haematoxyin and eosin (H&E) and Luxol fast blue (LFB) staining. Section slides were examined and imaged with a light microscope (Zeiss Axioscope). Histology scoring was performed following previous guidelines^[^ ^48^, ^73^ ^]^ by two researchers in a blinded manner.

### T Cell Polarization Experiment

Naïve CD4^+^ T cells purified from the spleen (>90% purity using the MACS Naïve CD4^+^ T cell Isolation Kit; Miltenyi Biotec) were seeded at a concentration of 100 000 cells per well into a 96 well round‐bottom plate in the presence of 2 µg ml^−1^ plate‐bound anti‐CD3 (Clone 37.51) and 1 µg ml^−1^ soluble anti‐CD28 (Clone 17A2). For Treg polarization, 5 ng ml^−1^ TGF‐β and 10 ng ml^−1^ IL‐2 were added to the culture. Cells were cultured for 5 days before quantification with flow cytometry.

### RNA Sequencing (RNA‐seq), Reduced Representation Bisulfite Sequencing (RRBS), and Single Cell RNA‐seq (scRNA‐seq)

Naïve CD4^+^ T cells were sorted from splenocytes using the Naive CD4^+^ T Cell Isolation Kit, mouse (Miltenyi Biotec, 130‐104‐453) to >95% purity. RNA and DNA extraction were performed using the AllPrep DNA/RNA/miRNA Universal Kit (Qiagen #80 224). RNA sequencing and multiplexed reduced representation bisulfite sequencing was done as previously described.^[^ ^93^ ^]^

For processing of RNA sequencing data, raw data was first quality filtered and processed using fastp v0.22.0, and sequences were aligned to the GRCm38 mouse reference genome using STAR v2.7.8a with two‐pass mapping. Gene count was quantified using HTSeq and genes with less than 10 counts across all samples were filtered out prior to analysis. Count data was the normalized with DESeq2^[^ ^94^ ^]^ and analyzed with Gene Set Enrichment Analysis (GSEA) software^[^ ^95^ ^]^ following their protocols.

For RRBS analysis, sequences were pre‐processed using fastp v0.22.0 for adapter trimming and quality filtering. Trimming of diversity adaptors were then performed using a custom script provided by the manufacturer (NuGEN). Data was then aligned to the GRCm39 reference genome using Bismark v0.22.1 and analyzed using methylKit. Differentially methylated regions were defined as those with a methylation signal difference larger than 10% and q value smaller than 0.05 between groups.

For scRNA‐seq analysis, data was downloaded from Gene Expression Omnibus (GSE199460 and GSE138266) and analyzed using Seurat^[^ ^96^, ^97^ ^]^ as described in the original publications. Gene set scores were calculated using the *CellCycleScoring* function in Seurat based on the corresponding gene sets described in GSEA.

### RNA Extraction and qPCR

Total RNA was extracted from spine tissue using TRI Reagent (Sigma‐Aldrich) based on the manufacturer's instructions. cDNA was then synthesized with the High‐Capacity cDNA Reverse Transcription Kit (ThermoFisher). QPCR was run with the Power SYBR Green PCR Master Mix (ThermoFisher) using a LightCycler 480 Instrument II (Roche). Gene expression was analyzed after normalization to housekeeping gene *Rpl13a*. primer sequences were listed in Table S13 (Supporting Information) and used at concentration of 200 nM.

### Mixture Modelling

Details of the analysis were described in the previous works.^[^ ^16^, ^42^ ^]^ In brief, impacts from dietary macronutrient compositions on outcomes could be analyzed with mixture models (also known as the Scheffe's polynomials), which was implemented using the mixexp package (1.2.5) in R. In the modelling, four models described by Lawson and Willden.^[^ ^70^ ^]^ and a null model would be fitted for the corresponding outcome, which reflected no effect, linear effects and non‐linear effects from the dietary macronutrient compositions. Each model was next evaluated based on the Akaike information criterion (AIC),^[^ ^98^ ^]^ and the one with the lowest AIC was selected as the best fitted model. Modelling results could be visualized on a right‐angled mixture triangle plot as previously described.

### Generalized Additive Mixed Models (GAMMs) Analysis

Data curation was previously described in ref. [36] In brief, multiple sclerosis disease burden data was obtained from the Global Burden of Disease Study 2019 (GBD2019). Gross domestic product (GDP) data was extracted from the Maddison project.^[^ ^99^ ^]^ Macronutrient supply data was from the Food and Agriculture Organization Corporate Statistical Dabase (FAOSTAT, www.fao.org/faostat/en/#home). Analyses were based on 1990–2018 and all data went through quality check to exclude countries and timepoints that had no record. Resulting data covering 1990–2018 for ≈150 countries were left for further analysis using R.

Details of GAMM analysis were described in Supporting Information and in the previous works.^[^ ^36^, ^37^, ^38^, ^40^ ^]^ In brief, since the non‐linear and interactive effects of dietary macronutrients are gaining more interests in nutritional research, significance of multi‐dimensional thinking was receiving more emphasis.^[^ ^36^, ^37^, ^38^, ^39^ ^]^ These could be achieved via the state‐of‐the‐art nutritional geometry GAMM analysis. GAMM was a multiple regression tool, based on similar assumptions to generalized linear models. GAMMs account for the non‐linear terms as non‐parametric smoothed functions, providing a flexible manner to estimate the non‐linear effects. Importantly, GAMMs could also adjust for a plethora of other confounders like GDP as a close proxy of socioeconomic status, in a similar way to conventional linear regression used in epidemiological studies.

Here, a series of GAMMs were fitted to model the influences from nutrient supplies on MS disease burden globally over time. Factors of time and the GDP of each country at each timepoint were considered as well and individual country the data originated was accounted as a random effect, which could adjust for some confounders among countries like differences in ethnicity and genetics. Using nutrient supply, year, and GDP data as predictors, multiple variable GAMMs considered all combinations of the individual, additive and interactive effects for all the predictors. Modelling outcomes were evaluated using AIC and the one with the lowest AIC was selected.

### Statistical Analysis

Data were statistically analyzed with PRISM GraphPad. A one‐way ANOVA was used when comparing three groups, and a two‐way ANOVA, with diets and time as parameters, was used to analyze the EAE clinical curves.

## Conflict of Interest

Laurence Macia is a current employee of the Translational Science Hub Global Sanofi Vaccines R&D Brisbane, Australia. Her contribution to this work was when she was an employee of the University of Sydney. The other authors declare no conflicts of interest.

## Author Contributions

L.M. funded, conceived and designed the studies. D.N. R.N. and L.M. wrote the manuscript. D.N. performed most of the experiments. J.T., J.R., J.T., C.P., C.W., A.S., and L.P. performed animal and lab experiments. A.M.S., C.A., D.R., and S.J.S. performed the nutritional geometry modelling analyses. R.B. performed the sequencing experiments. N.J.C.K. contributed to reading, editing and approving the manuscript. All authors read and approved the final manuscript.

## Supporting information



Supporting Information

## Data Availability

The data that support the findings of this study are available from the corresponding author upon reasonable request.
