# Supplementary material for: High Fat Low Carbohydrate Diet Is Linked to CNS Autoimmunity Protection
Source: Adv Sci (Weinh). 2025 Mar 27;12(22):2412236. doi: 10.1002/advs.202412236 (PMC12165102; doi:10.1002/advs.202412236)
Supplement: Supplementary file 1 — Supporting Information [file ADVS-12-2412236-s001.pdf]

ADVANCED SCIENCE
Open Access

Supporting Information

for *Adv. Sci.*, DOI 10.1002/advs.202412236

High Fat Low Carbohydrate Diet Is Linked to CNS Autoimmunity Protection

*Duan Ni, Jian Tan, Julen Reyes, Alistair M Senior, Caitlin Andrews, Jemma Taitz, Camille Potier-Villette, Claire Wishart, Alanna Spiteri, Laura Piccio, Nicholas Jonathan Cole King, Romain Barrès, David Raubenheimer, Stephen James Simpson, Ralph Nanan and Laurence Macia**

Figure S1.

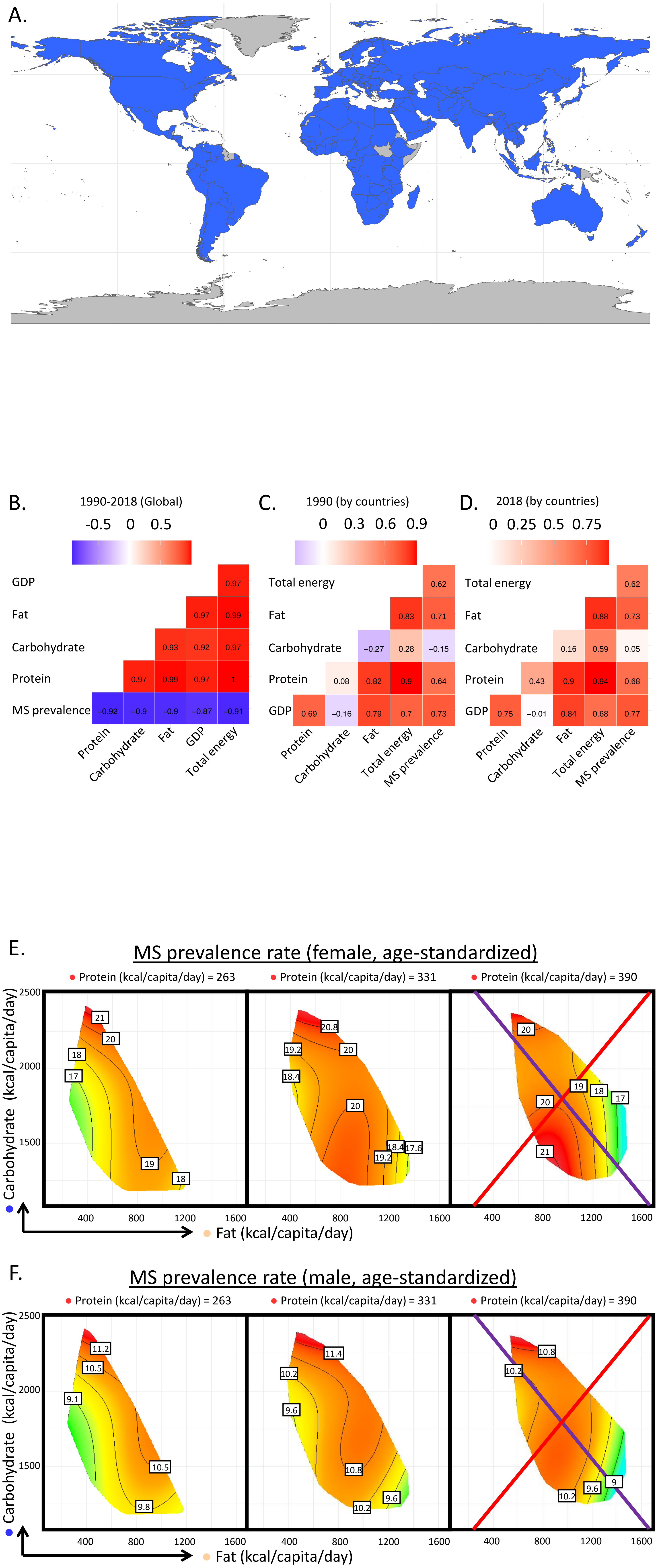
A.
B.
1990-2018 (Global)
-0.5 0 0.5
GDP 0.97
Fat 0.97 0.99
Carbohydrate 0.93 0.92 0.97
Protein 0.97 0.99 0.97 1
MS prevalence −0.92 −0.9 −0.9 −0.87 −0.91
Protein
Carbohydrate
Fat
GDP
Total energy
C.
1990 (by countries)
0 0.3 0.6 0.9
Total energy 0.62
Fat 0.83 0.71
Carbohydrate −0.27 0.28 −0.15
Protein 0.08 0.82 0.9 0.64
GDP 0.69 −0.16 0.79 0.7 0.73
Protein
Carbohydrate
Fat
Total energy
MS prevalence
D.
2018 (by countries)
0 0.25 0.5 0.75
Total energy 0.62
Fat 0.88 0.73
Carbohydrate 0.16 0.59 0.05
Protein 0.43 0.9 0.94 0.68
GDP 0.75 −0.01 0.84 0.68 0.77
Protein
Carbohydrate
Fat
Total energy
MS prevalence
E.
MS prevalence rate (female, age-standardized)
Protein (kcal/capita/day) = 263
Protein (kcal/capita/day) = 331
Protein (kcal/capita/day) = 390
Carbohydrate (kcal/capita/day)
Fat (kcal/capita/day)
F.
MS prevalence rate (male, age-standardized)
Protein (kcal/capita/day) = 263
Protein (kcal/capita/day) = 331
Protein (kcal/capita/day) = 390
Carbohydrate (kcal/capita/day)
Fat (kcal/capita/day)

Figure S2.

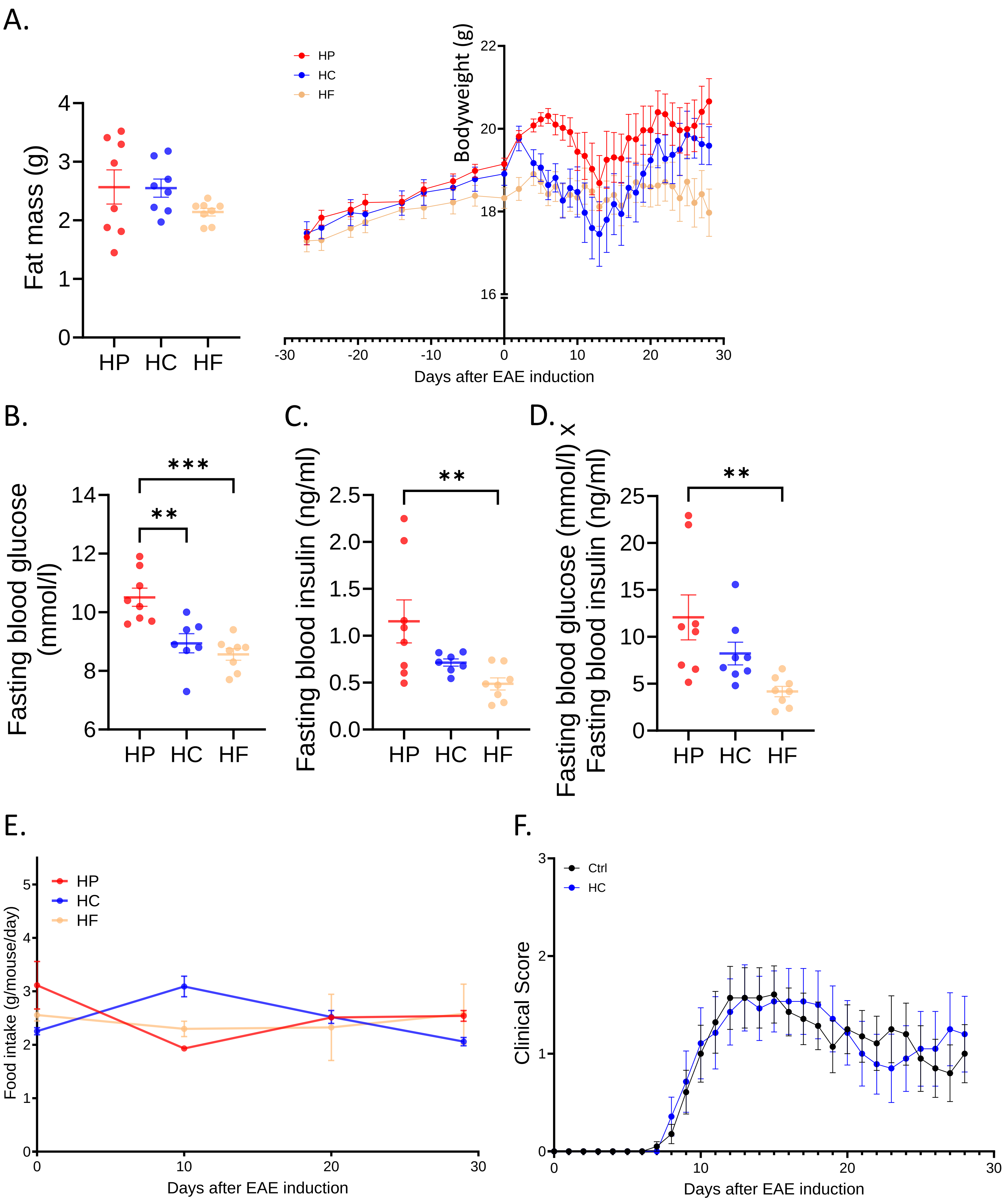
A.
Fat mass (g)
HP HC HF
HP
HC
HF
Bodyweight (g)
Days after EAE induction
B.
Fasting blood glucose (mmol/l)
***
**
C.
Fasting blood insulin (ng/ml)
**
D.
Fasting blood glucose (mmol/l) x Fasting blood insulin (ng/ml)
**
E.
Food intake (g/mouse/day)
Days after EAE induction
F.
Ctrl
HC
Clinical Score
Days after EAE induction

Figure S3.

A.

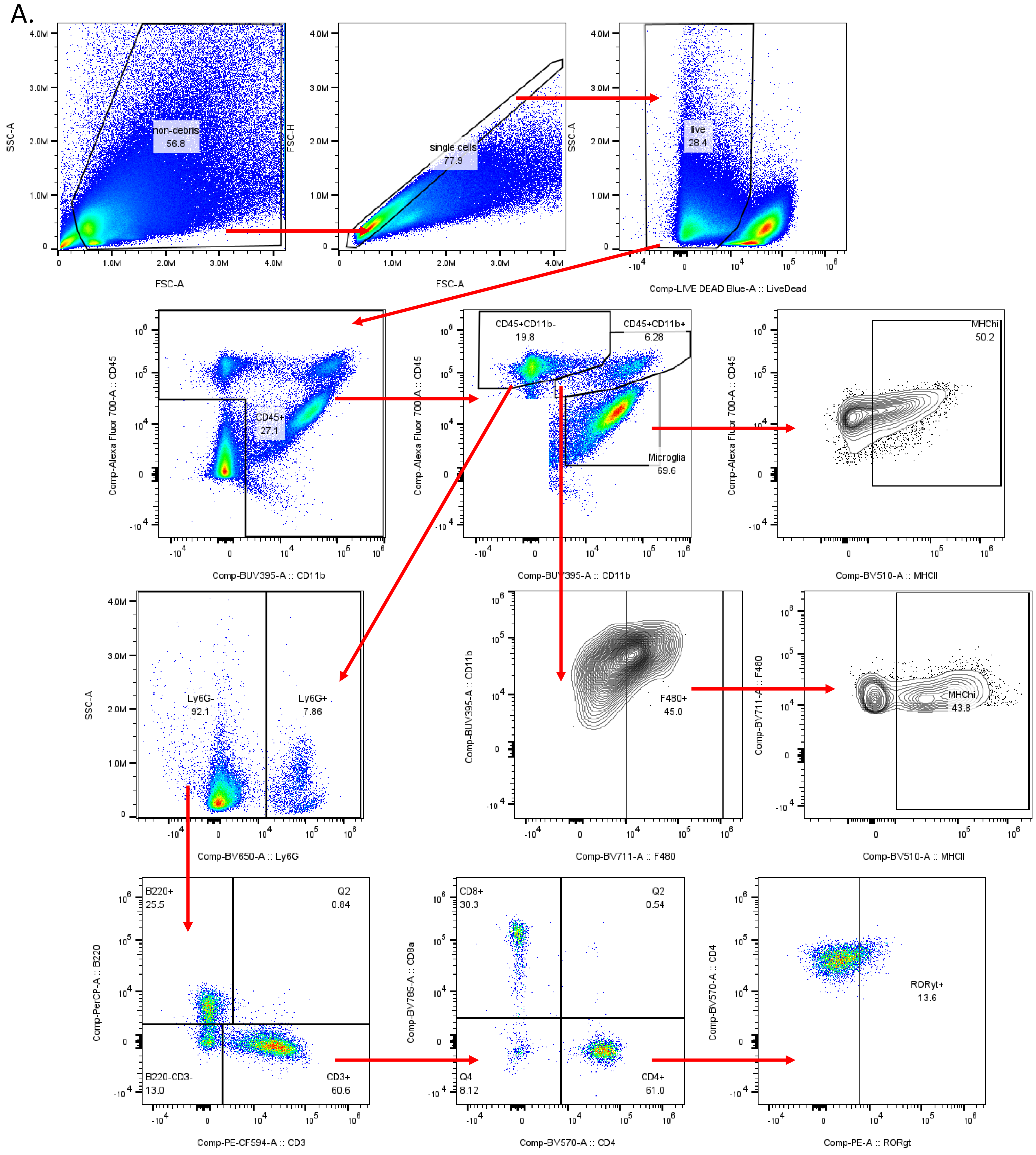

non-debris
56.8
SSC-A
FSC-A
single cells
77.9
FSC-H
live
28.4
Comp-LIVE DEAD Blue-A :: LiveDead
CD45+
27.1
Comp-Alexa Fluor 700-A :: CD45
Comp-BUV395-A :: CD11b
CD45+CD11b-
19.8
CD45+CD11b+
6.28
Microglia
69.6
MHChi
50.2
Comp-BV510-A :: MHCII
Ly6G-
92.1
Ly6G+
7.86
Comp-BV650-A :: Ly6G
F480+
45.0
Comp-BV711-A :: F480
MHChi
43.8
B220+
25.5
Q2
0.84
B220-CD3-
13.0
CD3+
60.6
Comp-PerCP-A :: B220
Comp-PE-CF594-A :: CD3
CD8+
30.3
Q2
0.54
Q4
8.12
CD4+
61.0
Comp-BV785-A :: CD8a
Comp-BV570-A :: CD4
RORyt+
13.6
Comp-PE-A :: RORgt

B.

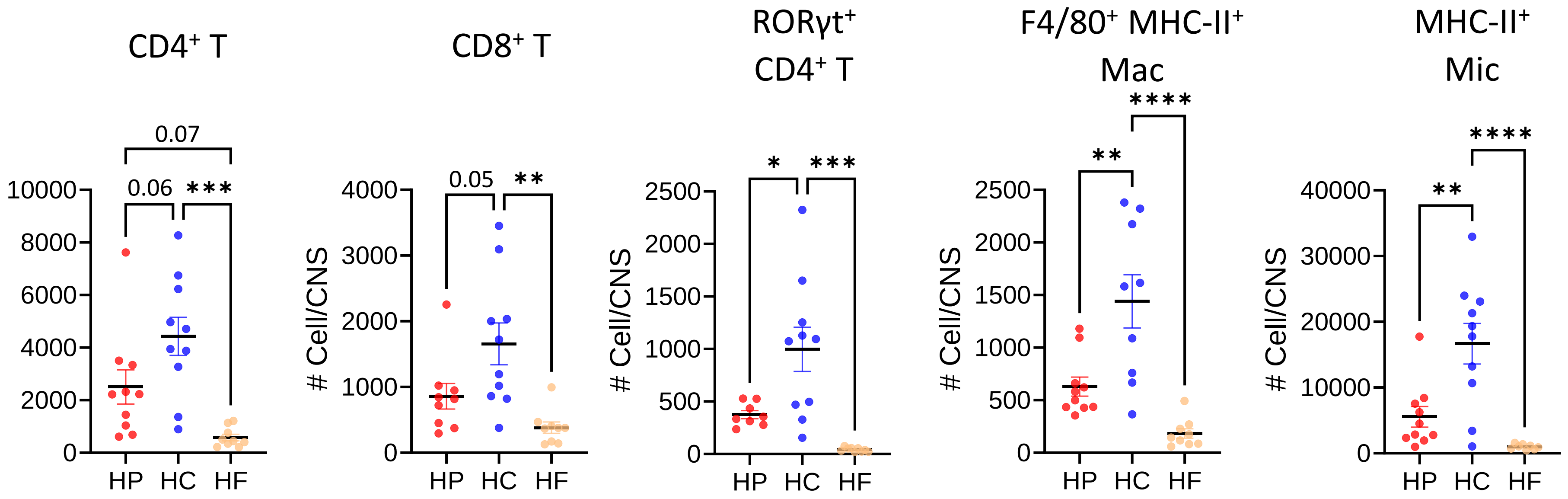

CD4+ T
CD8+ T
RORγt+ CD4+ T
F4/80+ MHC-II+ Mac
MHC-II+ Mic
Cell/CNS
HP
HC
HF
0.07
0.06
0.05

Figure S4.

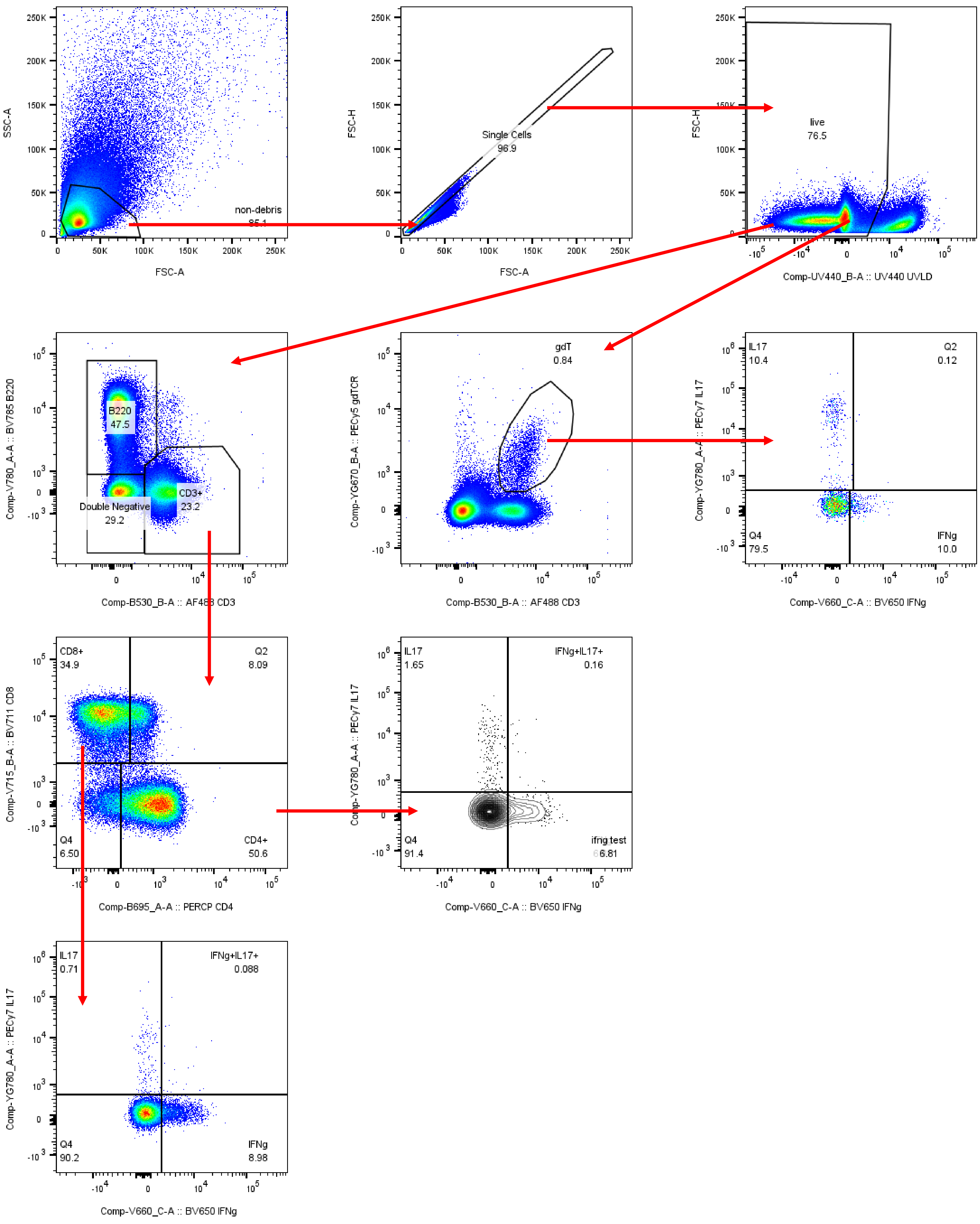
SSC-A
FSC-A
non-debris
FSC-H
Single Cells
96.9
live
76.5
Comp-UV440_B-A :: UV440 UVLD
Comp-V780_A-A :: BV785 B220
B220
47.5
Double Negative
29.2
CD3+
23.2
Comp-B530_B-A :: AF488 CD3
Comp-YG670_B-A :: PECy5 gdTCR
gdT
0.84
Comp-YG780_A-A :: PECy7 IL17
IL17
10.4
Q2
0.12
Q4
79.5
IFNg
10.0
Comp-V660_C-A :: BV650 IFNg
Comp-V715_B-A :: BV711 CD8
CD8+
34.9
Q2
8.09
Q4
6.50
CD4+
50.6
Comp-B695_A-A :: PERCP CD4
IL17
1.65
IFNg+IL17+
0.16
Q4
91.4
ifng test
6.81
IL17
0.71
IFNg+IL17+
0.088
Q4
90.2
IFNg
8.98

Figure S5.

A.

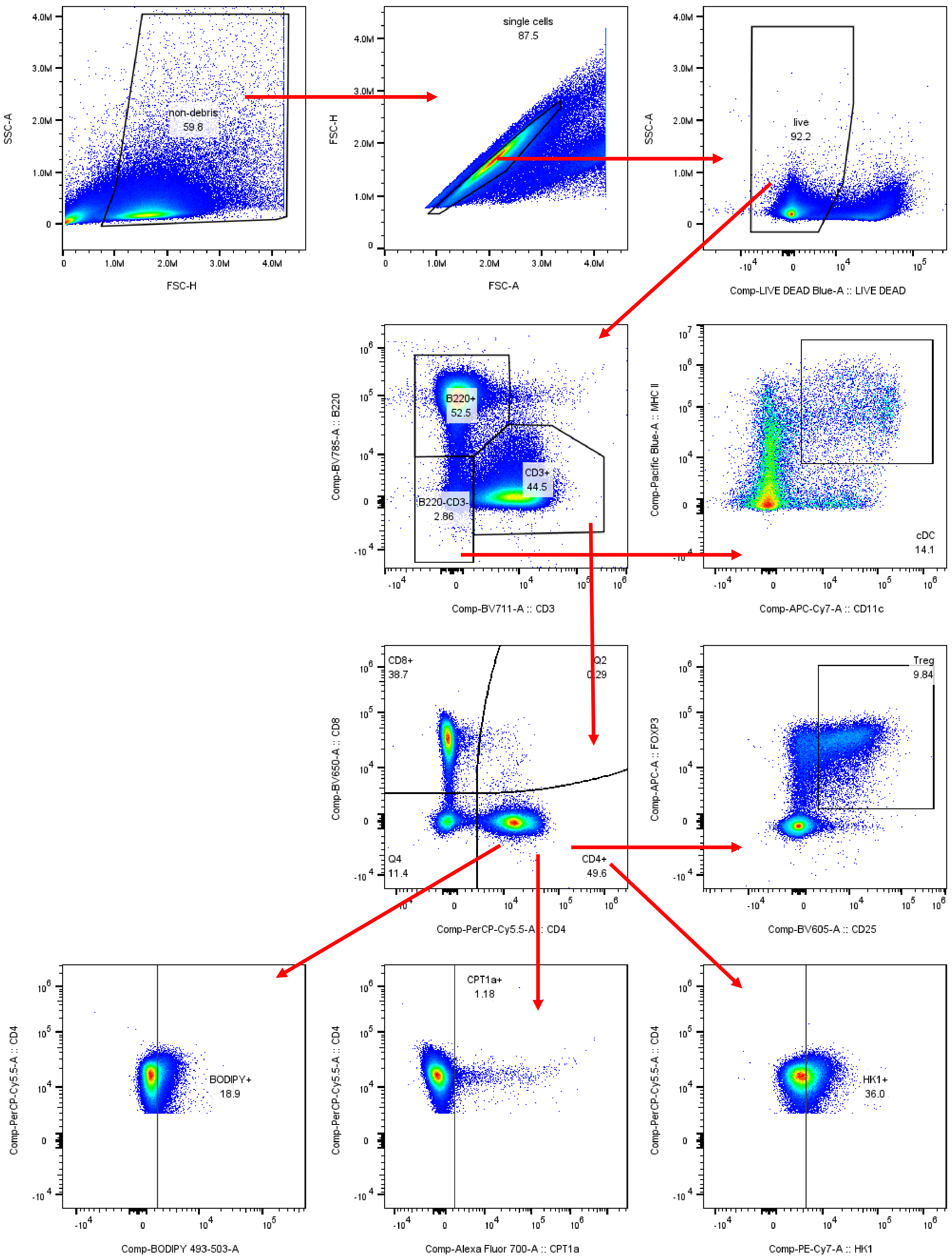

B.

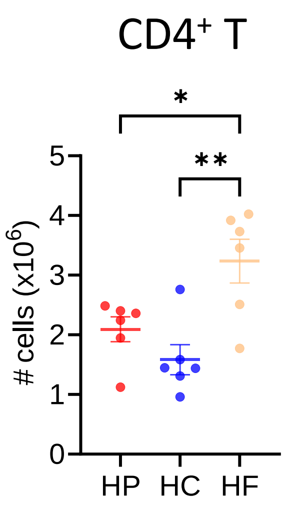

C.

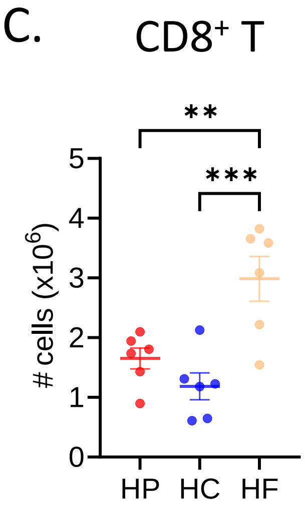

Figure S6.

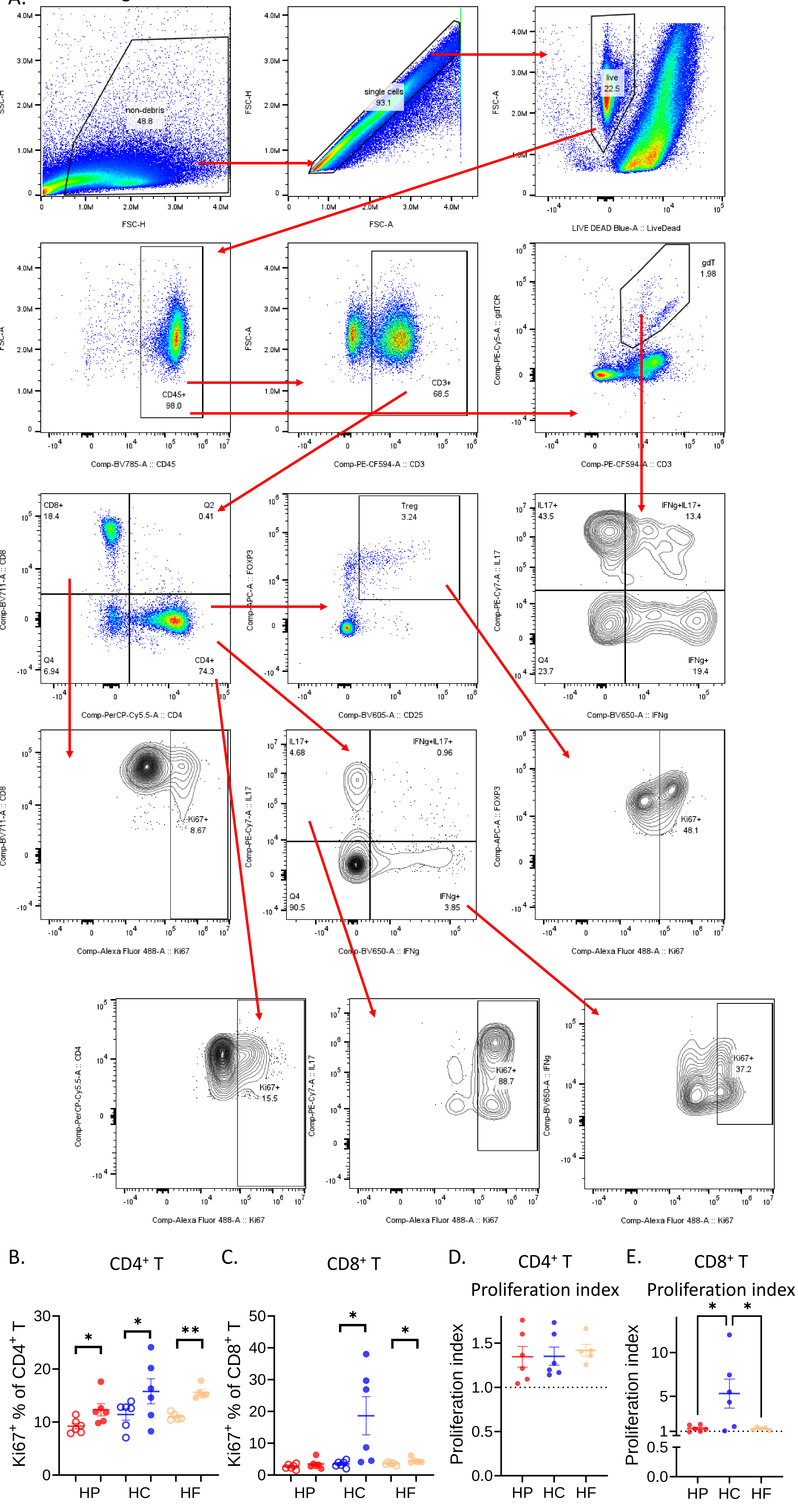
A.
non-debris
48.8
SSC-H
FSC-H
single cells
93.1
FSC-A
live
22.5
LIVE DEAD Blue-A :: LiveDead
CD45+
98.0
Comp-BV785-A :: CD45
CD3+
68.5
Comp-PE-CF594-A :: CD3
gdT
1.98
Comp-PE-Cy5-A :: gdTCR
CD8+
18.4
Q2
0.41
Q4
6.94
CD4+
74.3
Comp-BV711-A :: CD8
Comp-PerCP-Cy5.5-A :: CD4
Treg
3.24
Comp-APC-A :: FOXP3
Comp-BV605-A :: CD25
IL17+
43.5
IFNg+IL17+
13.4
Q4
23.7
IFNg+
19.4
Comp-PE-Cy7-A :: IL17
Comp-BV650-A :: IFNg
Ki67+
8.67
Comp-Alexa Fluor 488-A :: Ki67
IL17+
4.68
IFNg+IL17+
0.96
Q4
90.5
IFNg+
3.85
Ki67+
48.1
Ki67+
15.5
Ki67+
88.7
Ki67+
37.2
B.
CD4+ T
Ki67+ % of CD4+ T
HP
HC
HF
C.
CD8+ T
Ki67+ % of CD8+ T
D.
CD4+ T
Proliferation index
E.
CD8+ T
Proliferation index

Figure S7.

A.

GSE199460
Mouse EAE
Brain immune cells scRNA-seq

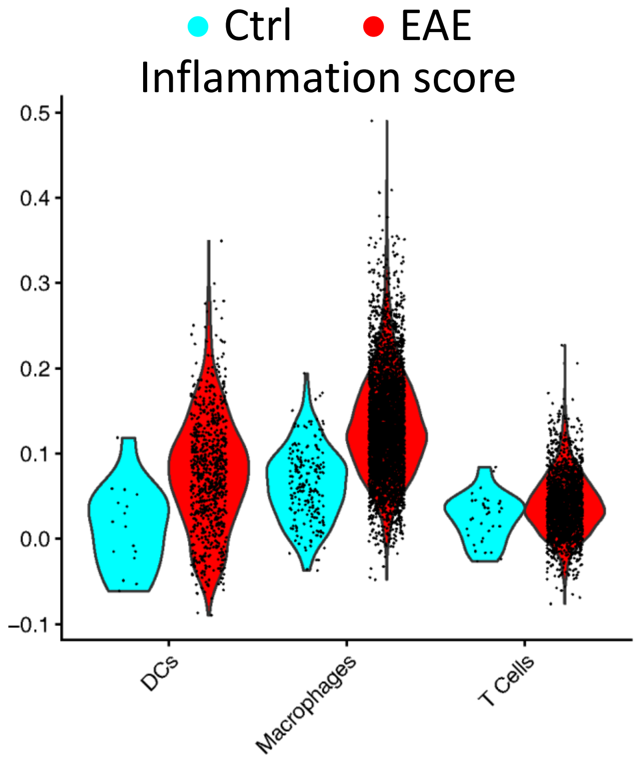

B.

GSE138266 Human MS PBMC scRNA-seq

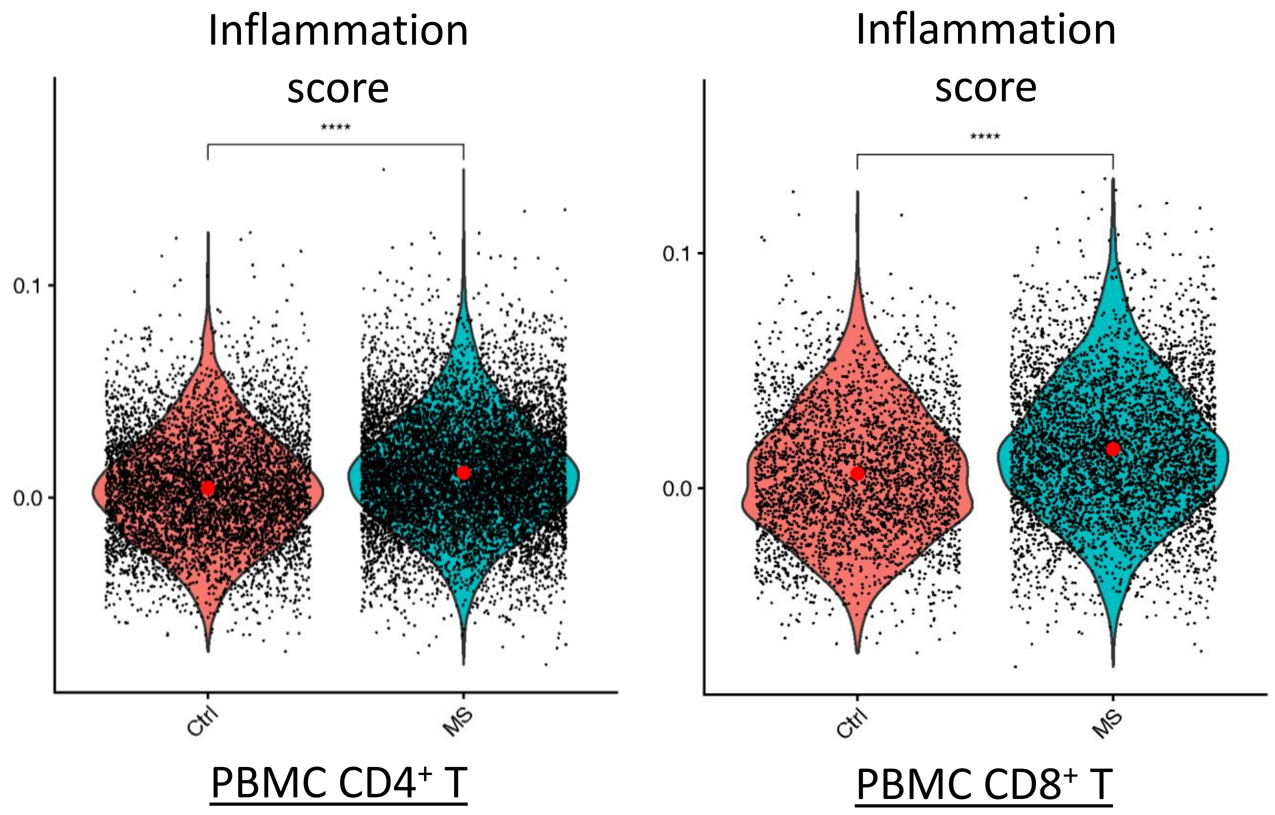

Figure S8.

A.

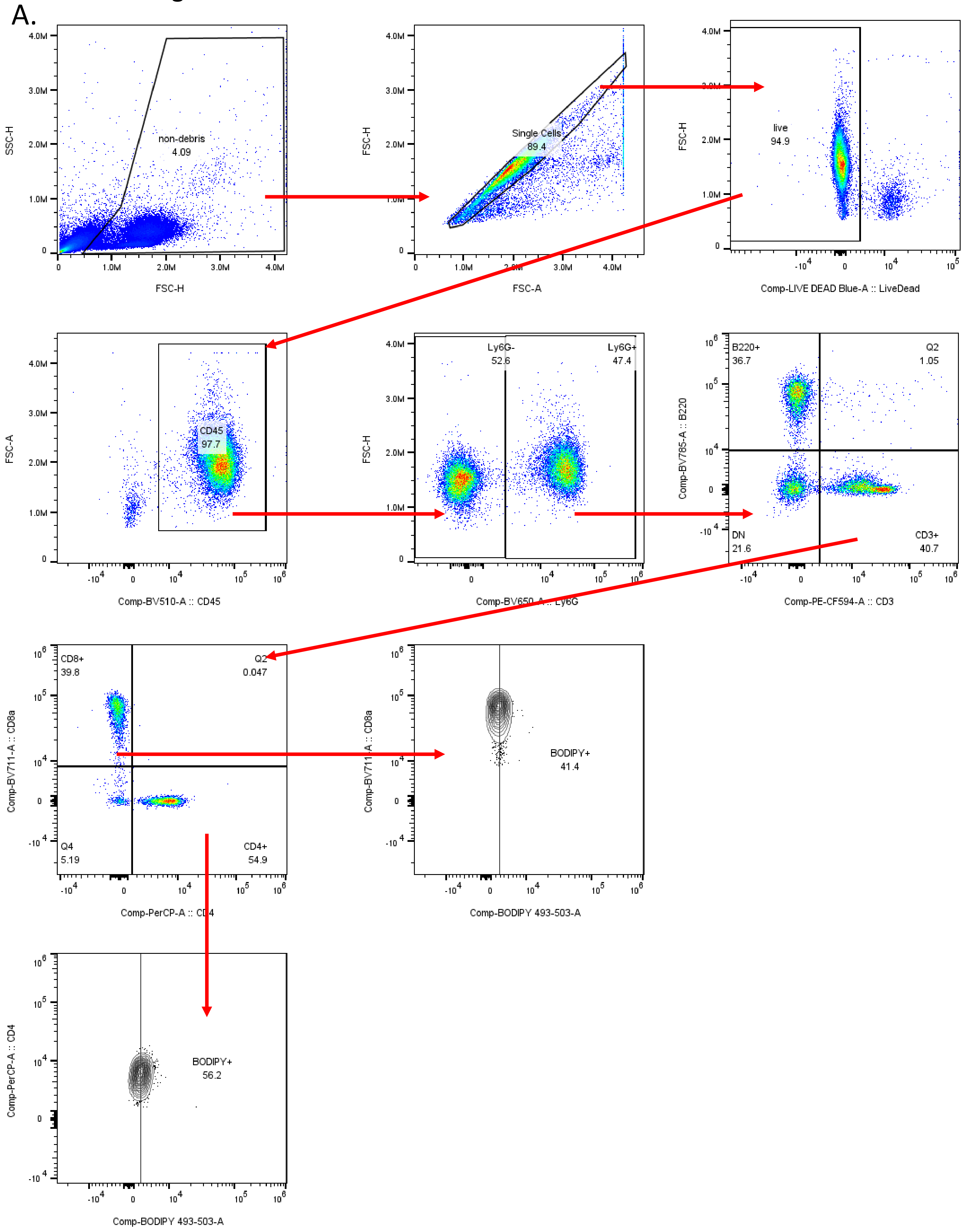

B.

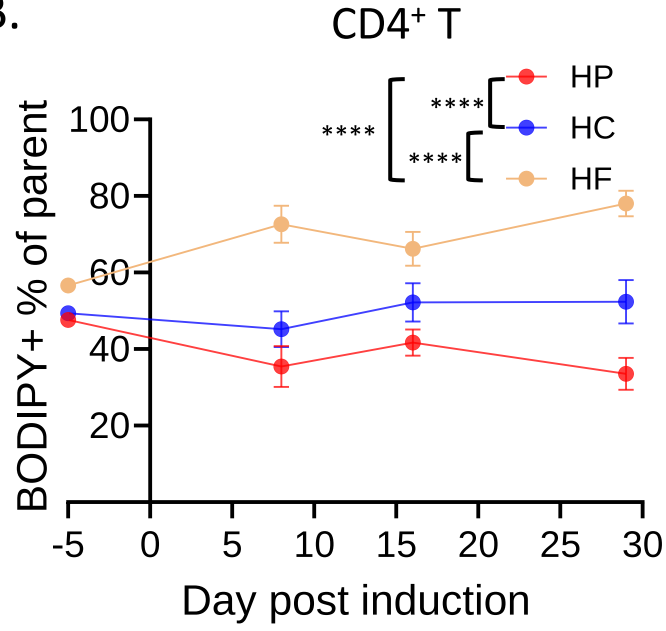

C.

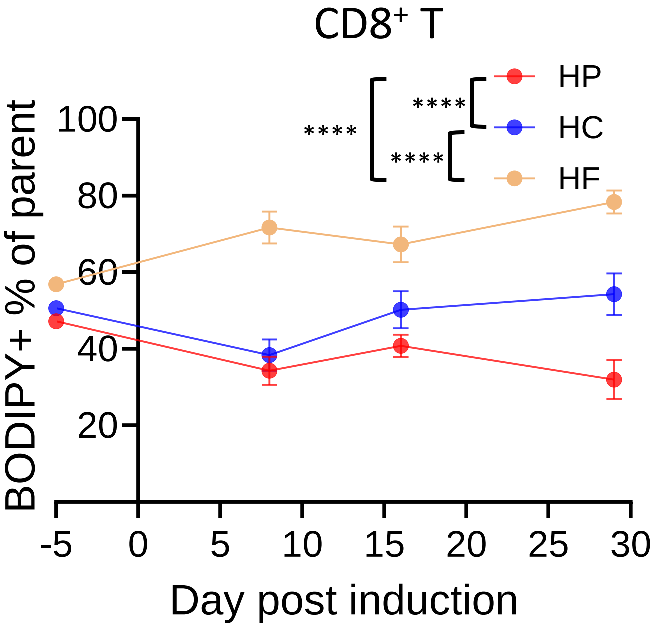

Figure S9.

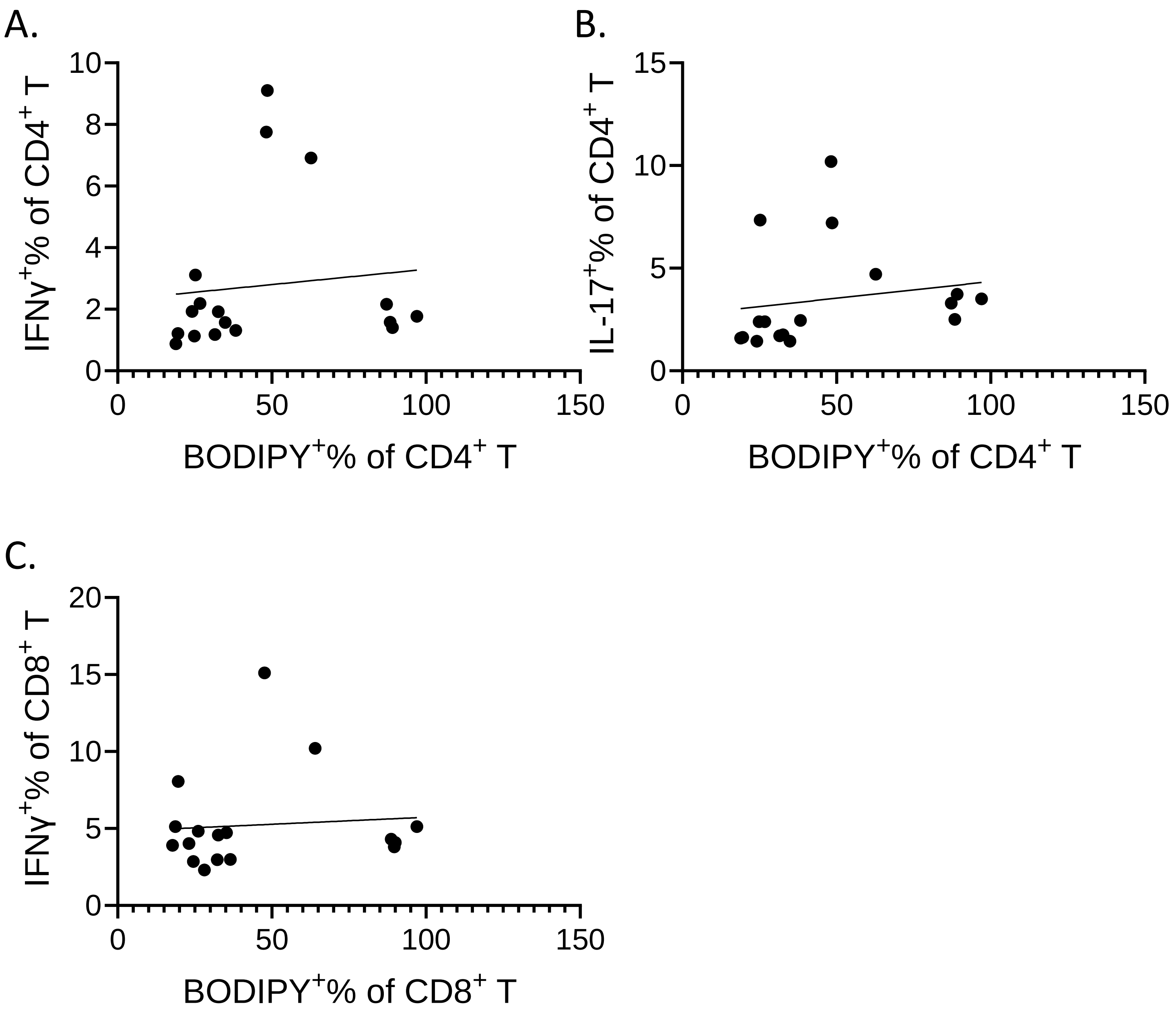
A.
IFNγ+% of CD4+ T
BODIPY+% of CD4+ T
B.
IL-17+% of CD4+ T
BODIPY+% of CD4+ T
C.
IFNγ+% of CD8+ T
BODIPY+% of CD8+ T

Figure S10.

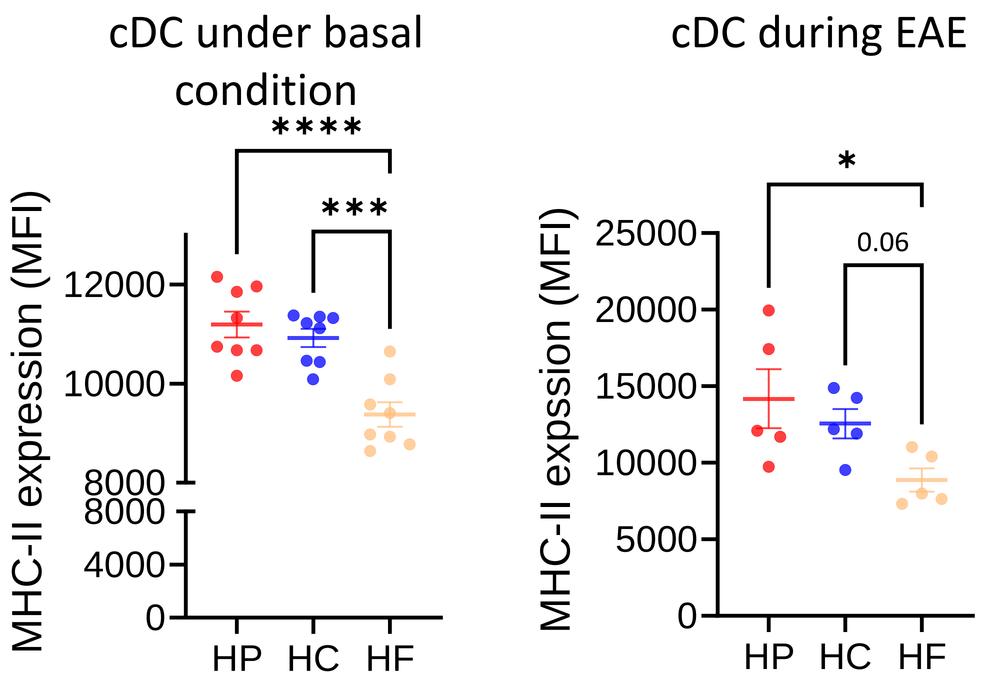

cDC under basal condition
****
***
MHC-II expression (MFI)
12000
10000
8000
8000
4000
0
HP
HC
HF
cDC during EAE
*
0.06
MHC-II expsion (MFI)
25000
20000
15000
10000
5000
0
HP
HC
HF

Figure S11.

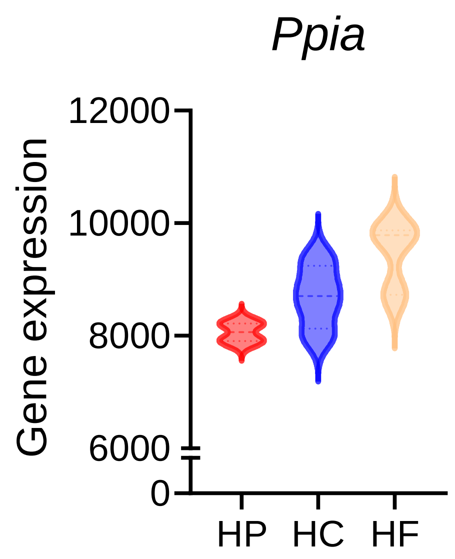

Supplementary Information

Interpretation of modelling surfaces

Details for modelling surface interpretation can refer to previous publications [1-3]. In brief, utilizing RStudio (v4.2.2.), multiple sclerosis (MS) disease burden data were analyzed with generalized additive mixed models (GAMMs) [4,5]. Analysis results of the supplies of three macronutrients were presented as response surfaces with focus on fat (*x*-axis) and carbohydrate (*y*-axis). The third macronutrient protein was controlled at 25%, 50% (median) and 75% quantiles of the global supply.

Within response surfaces, red means higher values, while blue denotes lower ones. Along the black contour lines, the modelled values are constant and the numbers on the lines denote the magnitude of the modelled parameters. The purple vector is an isocaloric line, along which the total energy supply from macronutrients is unchanged but fat is substituted for carbohydrate isocalorically. The red vector is a food rail. Carbohydrate:fat ratio is held unchanged along it, while the total macronutrient energy supply is altered.

Statistics for the modelling analysis are provided in Supplementary Tables. When the modelling analysis is significant, the effect of macronutrient supply on the modelling values (i.e., MS disease burden) can be deduced from the modelling surfaces.

Supplementary Methods

Data collection and processing

Data collation and processing were adapted from previous studies [1,3]. MS data was from the Global Burden of Disease Study 2019 (GBD 2019). As reported in [1], macronutrient supply data and gross domestic product (GDP) data were collected from the Food and Agriculture Organization Corporate Statistical Database (FAOSTAT, www.fao.org/faostat/en/#home) and the Maddison project [6] respectively.

Analyses were carried out on data from 1990 to 2018 with relatively comprehensive data coverage. Countries or time points with no record were filtered and the resulting data covering more than 150 countries, on all continents, were further analyzed with R (Figure S1A).

Generalized additive mixed models (GAMMs)

Details of GAMMs were described in [1,3]. In brief, GAMMs [4,7,8] were used to model the changes in MS burden over time and inspect the impacts from macronutrient supplies and GDP. GAMMs are based on assumptions similar to generalized linear models. GAMMs account for the nonlinear terms as nonparametric smoothed functions, often in a form of spline, and provide a flexible manner to estimate

the nonlinear associations. All modelling was run with the *mgcv* package and its “gam” function [4,5]. All models considered the country that the data were from as a random effect. The gamma parameter, implying the smoothing degrees of the modelled effects was defined as log(n)/2, where n is the number of combinations for countries and years with available data. A Gaussian family with log-link function was used for modelling.

Several different predictors and their different combinations as well as a null model where only the random effect from the country is considered are comparatively analyzed. Models with multiple variables consider all combinations of the individual, additive and interactions among parameters like macronutrient supply, year and GDP data. Macronutrient supply was modelled as a three-dimensional spline, and year and GDP data were modelled as one-dimensional cubic-regression splines based on the “s()” function in *mgcv* package. The interactions between smooth terms on different scales such as macronutrient supply and year were modelled with “te()” function from the package using tensor product smoothers.

Modelling results were compared using Akaike information criterions (AICs) and the model with the lowest AIC was selected [9].

Codes for the analysis are adapted from GitHub, https://github.com/Nidane/Asthma-NutrientSupply.

Document S1. Figure S1-11,

Document S2. Supplementary Information & Table S1-13.

Supplementary Tables

Supplementary Tables 1-4 are GAMMs estimates. For parametric terms the model estimates and associated standard errors (SE) and test statistics are presented. For non-parametric smooth terms, the estimated and reference degrees of freedom (reflected by edf, sumEDF, Ref.df) are shown as well as their test statistics. Smooth terms were fitter with either the standard smooth function “s()” or a tensor product smooth “te()” in the *mgcv* package. Tensor product smoothing was utilized where terms exist on different units (for example, nutrient supply and year). Country was included as a random effect using the smooth function “s()”. The model family and implemented link function is stated.

Table S1. Relative fit of generalized additive mixed models (GAMMs) testing the predictors for age-standardized MS prevalence rate in both sexes. Gamma is the degrees of freedom inflation factor. Dev means the deviance explained. AIC = Akaike information criterion. GDP = gross domestic product. Delta is the differences between AICs of models and the minimum AIC. sumEDF reflects the degrees of freedom of the models. Macronutrient supply was modelled as a three-dimensional thin-plate spline. (related to Figure 1C)

GAM	Gamma	Dev	AIC	Delta	Weights	sumEDF	Formula
1	3.855439	99.23836	21562.45	6238.577	0	155.7094	1 + s(Country, bs="re")
2	3.855439	99.3278	21024.99	5701.124	0	165.8786	s(protein.kcal, carb.kcal, fat.kcal, k=k_nut) + s(Country, bs="re")
3	3.855439	99.67816	17724.08	2400.208	0	159.6247	s(Year, k=10, bs="cr") + s(Country, bs="re")
4	3.855439	99.64768	18139.15	2815.286	0	165.1669	s(GDP, k=10, bs="cr") + s(Country, bs="re")
5	3.855439	99.70563	17344.83	2020.963	0	169.1888	s(protein.kcal, carb.kcal, fat.kcal, k=k_nut) + s(Year, k=10, bs="cr") + s(Country, bs="re")
6	3.855439	99.66184	17969.46	2645.588	0	171.9048	s(protein.kcal, carb.kcal, fat.kcal, k=k_nut) + s(GDP, k=10, bs="cr") + s(Country, bs="re")
7	3.855439	99.72071	17099.84	1775.974	0	164.1298	s(Year, k=10, bs="cr") + s(GDP, k=10, bs="cr") + s(Country, bs="re")
8	3.855439	99.73837	16844.83	1520.959	0	182.4681	te(protein.kcal, carb.kcal, fat.kcal, Year, bs=c("tp", "cr"), d=c(3,1), k=c(k_nut, 7)) + s(Country, bs="re")
9	3.855439	99.77511	16247.58	923.7075	2.62702465392377e-201	221.5802	te(protein.kcal, carb.kcal, fat.kcal, GDP, bs=c("tp", "cr"), d=c(3,1), k=c(k_nut, 7)) + s(Country, bs="re")
10	3.855439	99.76975	16279.05	955.183	3.84270111916248e-208	184.7612	te(Year, GDP, k=10) + s(Country, bs="re")
11	3.855439	99.77028	16278	954.1295	6.50743485689478e-208	189.3544	te(protein.kcal, carb.kcal, fat.kcal, Year, bs=c("tp", "cr"), d=c(3,1), k=c(k_nut, 7)) + s(GDP, k=10, bs="cr") + s(Country, bs="re")
**12**	**3.855439**	**99.81705**	**15323.87**	**0**	**1**	**220.6137**	**te(protein.kcal, carb.kcal, fat.kcal, GDP, bs=c("tp", "cr"), d=c(3,1), k=c(k_nut, 7)) + s(Year, k=10, bs="cr") + s(Country, bs="re")**
13	3.855439	99.79315	15817.69	493.8237	5.85512728584453e-108	193.3464	te(Year, GDP, k=10) + s(protein.kcal, carb.kcal, fat.kcal, k=k_nut) + s(Country, bs="re")

Table S2. Estimated effects of macronutrient supply by time and GDP per capita on age-standardized MS prevalence rate. Gaussian-GAMM, log-link function. (related to Figure 1C)

**Parametric coefficients**				
	**Estimate**	**Std. Error**	**t value**	**Pr(>|t|)**
**(Intercept)**	2.63997	0.07409	35.63	<2e-16
**Approximate significance of smooth terms**				
	**edf**	**Ref.df**	**F**	**p-value**
**te(protein.kcal,carb.kcal,fat.kcal,GDP)**	60.370	64.215	49.26	<2e-16
**s(Year)**	2.623	3.311	300.34	<2e-16
**s(Country)**	157.620	158.000	2419.12	<2e-16
R-sq.(adj) = 0.998 Deviance explained = 99.8%				
GCV = 2.5073 Scale est. = 1.7251 n = 4465				

Table S3. Relative fit of generalized additive mixed models (GAMMs) testing the predictors for age-standardized MS incidence rate in both sexes. Gamma is the degrees of freedom inflation factor. Dev means the deviance explained. AIC = Akaike information criterion. GDP = gross domestic product. Delta is the differences between AICs of models and the minimum AIC. sumEDF reflects the degrees of freedom of the models. Macronutrient supply was modelled as a three dimensional thin-plate spline. (related to Figure 1D)

GAM	Gamma	Dev	AIC	Delta	Weights	sumEDF	Formula
1	3.855439	99.40662	-9236.21	5144.647	0	156.7532	1 + s(Country, bs="re")
2	3.855439	99.44874	-9545.85	4835.011	0	166.3358	s(protein.kcal, carb.kcal, fat.kcal, k=k_nut) + s(Country, bs="re")
3	3.855439	99.61815	-11198.2	3182.612	0	159.8265	s(Year, k=10, bs="cr") + s(Country, bs="re")
4	3.855439	99.67643	-11925.4	2455.451	0	166.0184	s(GDP, k=10, bs="cr") + s(Country, bs="re")
5	3.855439	99.66642	-11781	2599.813	0	170.1413	s(protein.kcal, carb.kcal, fat.kcal, k=k_nut) + s(Year, k=10, bs="cr") + s(Country, bs="re")
6	3.855439	99.70085	-12256.9	2123.923	0	175.4444	s(protein.kcal, carb.kcal, fat.kcal, k=k_nut) + s(GDP, k=10, bs="cr") + s(Country, bs="re")
7	3.855439	99.70625	-12350.9	2029.936	0	169.0914	s(Year, k=10, bs="cr") + s(GDP, k=10, bs="cr") + s(Country, bs="re")
8	3.855439	99.72286	-12577	1803.83	0	185.9476	te(protein.kcal, carb.kcal, fat.kcal, Year, bs=c("tp", "cr"), d=c(3,1), k=c(k_nut, 7)) + s(Country, bs="re")
9	3.855439	99.79998	-13954.4	426.4525	2.49473200555675e-93	225.2808	te(protein.kcal, carb.kcal, fat.kcal, GDP, bs=c("tp", "cr"), d=c(3,1), k=c(k_nut, 7)) + s(Country, bs="re")
10	3.855439	99.76006	-13218.2	1162.679	3.36881050823119e-253	187.165	te(Year, GDP, k=10) + s(Country, bs="re")
11	3.855439	99.76936	-13383.8	997.0757	3.07448772474725e-217	192.6561	te(protein.kcal, carb.kcal, fat.kcal, Year, bs=c("tp", "cr"), d=c(3,1), k=c(k_nut, 7)) + s(GDP, k=10, bs="cr") + s(Country, bs="re")
**12**	**3.855439**	**99.81843**	**-14380.9**	**0**	**1**	**228.1745**	**te(protein.kcal, carb.kcal, fat.kcal, GDP, bs=c("tp", "cr"), d=c(3,1), k=c(k_nut, 7)) + s(Year, k=10, bs="cr") + s(Country, bs="re")**
13	3.855439	99.78634	-13719.4	661.4657	2.31501465953139e-144	195.5588	te(Year, GDP, k=10) + s(protein.kcal, carb.kcal, fat.kcal, k=k_nut) + s(Country, bs="re")

Table S4. Estimated effects of macronutrient supply (plant- and animal-based fats and carbohydrate and protein) by time and GDP per capita on age-standardized MS incidence rate. Gaussian-GAMM, log-link function. (related to Figure 1D)

**Parametric coefficients**				
	**Estimate**	**Std. Error**	**t value**	**Pr(>|t|)**
**(Intercept)**	-0.40866	0.07832	-5.218	1.9e-07
**Approximate significance of smooth terms**				
	**edf**	**Ref.df**	**F**	**p-value**
**te(pbf.kcal,carb_prot.kcal,abf.kcal,GDP)**	66.751	69.768	64.87	<2e-16
**s(Year)**	3.566	4.447	94.71	<2e-16
**s(Country)**	157.857	158.000	2746.79	<2e-16
R-sq.(adj) = 0.998 Deviance explained = 99.8%				
GCV = 0.0032772 Scale est. = 0.0022226 n = 4465				

Table S5. Detailed compositions of diets used in the current study.

	**Diet#**	**1 (HP)**	**2 (HC)**	**3 (HF)**	**4**	**5**	**6**	**7**	**8**	**9**	**10**
	**%Protein**	60	5	5	33	33	5	14	14	42	24
	**%Carbohydrate**	20	75	20	47	20	47	29	57	29	38
	**%Fat**	20	20	75	20	47	48	57	29	29	38
**Ingredients**											
**Protein**	**Casein**	64.87	5.04	5.04	35.50	35.50	5.04	14.83	14.83	45.29	25.71
	**L-Methionine**	0.30	0.30	0.30	0.30	0.30	0.30	0.30	0.30	0.30	0.30
**Fat**	**Canola oil**	7.91	7.91	29.65	7.91	18.58	18.98	22.53	11.46	11.46	15.03
**Carbohydrate**	**Wheat starch**	12.59	47.87	12.60	29.91	12.60	29.91	18.37	36.34	18.37	24.13
	**Dextrinized starch**	4.10	15.57	4.10	9.73	4.10	9.73	5.98	11.82	5.98	7.85
	**Sucrose**	3.11	11.81	3.11	7.38	3.11	7.38	4.54	8.97	4.54	5.96
**Minerals**	**$CaCO_3$**	1.31	1.31	1.31	1.31	1.31	1.31	1.31	1.31	1.31	1.31
	**NaCl**	0.26	0.26	0.26	0.26	0.26	0.26	0.26	0.26	0.26	0.26
	**AIN93 trace minerals**	0.14	0.14	0.14	0.14	0.14	0.14	0.14	0.14	0.14	0.14
	**KH_2PO_4**	0.69	0.69	0.69	0.69	0.69	0.69	0.69	0.69	0.69	0.69
	**KCl**	0.25	0.25	0.25	0.25	0.25	0.25	0.25	0.25	0.25	0.25
	**$C_5H_{14}ClNO$**	0.25	0.25	0.25	0.25	0.25	0.25	0.25	0.25	0.25	0.25
**Vitamins**	**AIN93 vitamins**	1.00	1.00	1.00	1.00	1.00	1.00	1.00	1.00	1.00	1.00
**Cellulose**	**Cellulose**	3.06	7.44	41.15	5.21	21.75	24.60	29.39	12.23	10.00	16.97

Table S6. Comparison of EAE clinical characteristics among diet groups (related to Figure 2B).

Exp. #	Group	N	Incidence	Mortality	Maximum clinical score (mean ± SEM)	Cumulative clinical score (mean ± SEM)
1	HP	10	5/10	0/10	1.35 ± 0.466	13.2 ± 5.465
	HC	10	9/10	0/10	2.5 ± 0.316	24.7 ±6.373
	HF	10	0/10	0/10	0 ± 0.000	0 ± 0.000
2	HP	10	5/10	0/10	0.95 ± 0.337	9.15 ± 4.701
	HC	10	6/10	0/10	1.8 ± 0.512	18.30 ± 5.214
	HF	9	0/9	0/9	0 ± 0.000	0 ± 0.000

Table S7. Significantly differential methylated genes revealed by reduced-representation bisulfite sequencing (RRBS-seq) in the HC vs HF and HP vs HF comparisons of their naïve $CD4^+$ T cells (genes that are consistently changed in both comparisons are in italic).

**HC vs HF, hypo-methylated in HF**						
**Gene name**	**chromosome**	**start**	**end**	**pvalue**	**qvalue**	**meth.diff**
Map3k11	*19*	*5688157*	*5689048*	*1.263595e-07*	*4.325089e-05*	*-15.22791*
Ankrd63	2	118702419	118704307	5.274150e-07	1.083156e-04	-15.01813
Ehmt2	17	34898533	34898905	3.498846e-05	3.421717e-03	-16.23913
H2-DMa	17	34118396	34122981	0.000282444	0.01611268	-13.70873
Abhd11	5	135008866	135009832	0.000480653	0.02223682	-13.10525
Tmem115	9	107534955	107535276	0.000716674	0.02777054	-10.65919
**HP vs HF, hypo-methylated in HF**						
**Gene name**	**chromosome**	**start**	**end**	**pvalue**	**qvalue**	**meth.diff**
Map3k11	*19*	*5688157*	*5689048*	*5.544530e-10*	*5.114604e-07*	*14.74388*
Thap3	4	151988595	151989048	1.010012e-06	1.164619e-04	12.05888
Gene not found	16	18234755	18235310	1.350604e-06	1.384309e-04	10.79941
Zbtb7a	10	81136019	81138167	1.709176e-05	9.274389e-04	11.61782
Tor2a	2	32757063	32757506	1.404853e-04	3.940120e-03	11.06557
Dusp3	11	101984403	101984969	2.372009e-04	5.209720e-03	12.08543
ADCK3	1	180195709	180196322	2.771312e-03	2.762718e-02	11.95767
Gpr153	4	152273871	152274518	4.358303e-03	3.717821e-02	10.83528
**HC vs HF, hyper-methylated in HF**						
**Gene name**	**chromosome**	**start**	**end**	**pvalue**	**qvalue**	**meth.diff**
Ube2q1	*3*	*89773101*	*89774061*	*3.581530e-07*	*8.179659e-05*	*18.15634*
Dock1	7	134670488	134671284	1.688617e-05	1.735267e-03	17.97599
8430429K09Rik	11	3452179	3452683	2.820396e-04	1.611268e-02	18.51328
Abl2	1	156558496	156559438	0.000740608	0.0278713	14.04762
Naa35	13	59585157	59585869	0.000747715	0.0278713	10.29173
**HP vs HF, hyper-methylated in HF**						
**Gene name**	**chromosome**	**start**	**end**	**pvalue**	**qvalue**	**meth.diff**
Cryba2	1	74892725	74893136	5.764289e-03	4.376398e-02	-11.34985

1110018N20Rik	2	167188125	167190391	2.171432e-06	1.907675e-04	-10.87842
Zfp777	6	48063944	48064147	2.586391e-05	1.223508e-03	-16.82738
Tollip	7	141902120	141902599	2.567512e-05	1.223508e-03	-14.35223
Ube2q1	*3*	*89773101*	*89774061*	*2.723055e-05*	*1.255954e-03*	*-11.36104*
Gm35025	9	108827073	108828002	6.041846e-04	1.032103e-02	-15.41029
Gm36794	5	111957837	111958080	2.177028e-03	2.449049e-02	-12.51291

Table S8. Statistical output for mixture models for mLN Treg (related Figure 4M).

**Model (Scheffe polynomials)**	**Akaike information criterion (AIC)**	**Degree of freedom**
**1**	259.619	4
**2**	261.497	7
**3**	267.014	11
**4**	261.632	8

**Model 1 coefficients**			
**Components**	**Estimate (Std.Error)**	**t value**	**P (>|t|)**
**Protein**	11.3591 (0.9644)	11.78	<2e-16
**Fat**	14.2565 (0.7298)	19.53	<2e-16
**Carbohydrate**	11.8080 (16.37)	16.37	<2e-16
**Adjusted R^2**	0.983		
**P value**	<2e-16		

Table S9. Statistical output for mixture models for thymus Treg (related Figure 4N).

**Model (Scheffe polynomials)**	**Akaike information criterion (AIC)**	**Degree of freedom**
**1**	98.132	4
**2**	102.813	7
**3**	105.749	11
**4**	104.779	8

**Model 1 coefficients**			
**Components**	**Estimate (Std.Error)**	**t value**	**P (>|t|)**
**Protein**	1.3054 (0.2162)	6.038	5.14e-08
**Fat**	1.4449 (0.1787)	8.085	6.97e-12
**Carbohydrate**	1.1967 (0.1804)	6.632	4.10e-09

**Adjusted R^2**	0.9022
**P value**	<2.2e-16

Table S10. Statistical output for mixture models for Treg polarization (related Figure 4O).

**Model (Scheffe polynomials)**	**Akaike information criterion (AIC)**		**Degree of freedom**
**1**	724.961		4
**2**	728.561		7
**3**	733.434		11
**4**	728.966		8
**Model 1 coefficients**			
**Components**	**Estimate (Std.Error)**	**t value**	**P (>|t|)**
**Protein**	63.967 (10.871)	5.884	9.80e-08
**Fat**	73.344 (8.986)	8.162	4.96e-12
**Carbohydrate**	65.589 (9.073)	7.229	3.06e-10
**Adjusted R^2**	0.9071		
**P value**	<2.2e-16		

Table S11. Key resource table.

**Reagent**	**Source**	**Identifier**
**Antibodies**		
Anti-mouse CD11b BUV395 (Clone M1/70)	BD	Cat# 565976; RRID: AB_2738276
Anti-mouse CD45 AF700 (Clone 30-F11)	BioLegend	Cat# 103128; RRID: AB_103128
Anti-mouse MHC-II BV510 (Clone 2G9)	BD	Cat# 743871; RRID: AB_1134102
Anti-mouse Ly6G BV650 (Clone 1A8)	BioLegend	Cat# 127641; RRID: AB_2565881
Anti-mouse F/480 BV711 (Clone BM8)	BioLegend	Cat# 123147; RRID: AB_2564588
Anti-mouse CD3 PE/CF594 (Clone 145-2C11)	BD	Cat# 562286; RRID: AB_11153307
Anti-mouse B220 PerCP (Clone RA3-6B2)	BioLegend	Cat# 1003234; RRID: AB_893353

Anti-mouse CD4 BV570 (Clone RM4-5)	BioLegend	Cat# 100542; RRID: AB_2563501
Anti-mouse CD8a BV785 (Clone 53-6.7)	BioLegend	Cat# 100750; RRID: AB_2562610
Anti-mouse RORγt PE	eBioscience	Cat# 12-6981-80; RRID: AB_10807092
Anti-mouse CD3 AF488 (Clone 17A2)	BioLegend	Cat# 100210; RRID: AB_389301
Anti-mouse B220 BV785 (Clone RA3-6B2)	BioLegend	Cat# 103245; RRID: AB_2563256
Anti-mouse γδTCR PE/Cy5 (Clone GL3)	eBioscience	Cat# 15-5711-82; RRID: AB_468804
Anti-mouse IFNγ BV650 (Clone XMG1.2)	BioLegend	Cat# 505832; RRID: AB_2734492
Anti-mouse IL-17 PE/Cy7 (Clone TC11-18H10.1)	BioLegend	Cat# 506922; RRID: AB_2125010
Anti-mouse CD4 PerCP (Clone RM4-5)	BioLegend	Cat# 100538; RRID: AB_893325
Anti-mouse CD8a BV711 (Clone 53-6.7)	BioLegend	Cat# 100759; RRID: AB_2563510
Anti-mouse CD3 BV711 (Clone 17A2)	BioLegend	Cat# 100241; RRID: AB_2563945
Anti-mouse CD11c APC/Cy7 (Clone N418)	BioLegend	Cat# 117324; RRID: AB_830649
Anti-mouse MHC-II Pacific Blue (Clone M5/114.15.2)	BioLegend	Cat# 107620; RRID: AB_493527
Anti-mouse CD4 PerCP/Cy5.5 (Clone GK1.5)	BioLegend	Cat# 100434; RRID: AB_2563056
Anti-mouse CD25 BV605 (Clone PC61)	BD	Cat# 563061; RRID: AB_2737982
Anti-mouse FOXP3 APC (Clone REA788)	Miltenyi	Cat# 130-111-601; RRID: AB_2651770
Anti-mouse Ki67 AF488 (Clone 16A8)	BioLegend	Cat# 652418; RRID: AB_ 2564269
**Chemicals and peptides**		
RPMI-1640	Thermo Fisher Scientific	Cat# 21870092
Foetal Bovine Serum	Bovogen Biologicals	Cat# AFBS-500
Phosphate-buffered saline (PBS)	Thermo Fisher Scientific	Cat# 18912014
Myelin oligodendrocyte glycoprotein 35-55 (MEV GWY RSP FSR VVH LYR NGK)	GenScript	Cat# 16913-87-9
Incomplete Freund's adjuvant (IFA)	Chondrex, Inc.	Cat# 7002
Pertussis toxin from *Bordetella pertussis*	Sigma	Cat# P7208
Phorbol 12-myristate 13-acetate	Sigma-Aldrich	Cat# P8139
Ionomycin calcium salt from Streptomyces conglobatus	Sigma-Aldrich	Cat# I0634
Brefeldin A	BioLegend	Cat# 420601

Penicillin/Streptomycin	Sigma-Aldrich	Cat# 15140122
HEPES buffer	Thermo Fisher Scientific	Cat# 15630080
LIVE/DEAD^{TM} Fixable Blue Dead Cell Stain Kit	Thermo Fisher Scientific	Cat# L34962
**Deposited data**		
GSE199460	scRNA-seq of EAE mouse CNS	
GSE235357	RNA-seq for MS patient PBMCs	
GSE138266	scRNA-seq for MS patient blood and cerebrospinal fluid leukocytes	
**Experimental models: Organisms**		
Mus muculus (C57BL/6)	Australian BioResources	JAX:000664
Mycobacterium tuberculosis Des. H37 Rα	BD	Cat# 231141

Table S12. Scoring guideline for the experimental autoimmune encephalomyelitis (EAE) clinical signs (related to Figure 2B).

EAE clinical score	Clinical signs
**0**	No signs of disease
**1**	Loss of tail tonus
**2**	Hind limb weakness
**3**	Hind limb paralysis
**4**	Paralysis of one forelimb
**5**	Paralysis of both forelimbs, moribund or dead

Table S13. List of primers used in qPCR assays (related to Figure 2D).

Gene	***Forward primer (5'-3')***	***Reverse primer (5'-3')***
Ifnγ	CGGCACAGTCATTGAAAGCC	TGTCACCATCCTTTTGCCAGT
Tnfa	ATGGCCTCCCTCTCATCAGT	GTTTGCTACGACGTGGGCTA
Nlrp3	GATGCTGGAATTAGACAACTG	GTACATTTCACCCAACTGTAG
Ccl2	CAAGATGATCCCAATGAGTAG	TTGGTGACAAAAACTACAGC
Cd68	CCAATTCAGGGTGGAAGAAA	CTCGGGCTCTGATGTAGGTC
Rpl13a	ATCCCTCCACCCTATGACAA	GCCCCAGGTAAGCAAACTT

Supplementary Figure Legends

Figure S1. A. An overview of the countries covered in the global analysis of macronutrient supplies and MS disease burden in 2018. **B-D.** Correlations for global variables from 1990 to 2018 (**B**) and for variables among different countries within 1990 (**C**) and 2018 (**D**). **E-F.** Predicted effects of macronutrient supply on age-standardized multiple sclerosis (MS) prevalence rate of female (**E**) and male (**F**).

Figure S2. A. Fat mass and bodyweight changes of mice fed on high protein (HP, red), high carbohydrate (HC, blue) and high fat (HF, light yellow) diets throughout the experiment. The day when experimental autoimmune encephalomyelitis (EAE) was induced was denoted as D0. **B-D.** Fasting blood glucose (mmol/l) (**B**), fasting blood insulin (ng/ml) (**C**) and their product (**D**) of mice after 6 weeks on HP, HC, and HF diets. **E.** Food intake of mice (g/mouse/day) fed on HP, HC and HF diets during EAE. **F.** EAE clinical curves of mice fed on AIN93G diet, the control (Ctrl) diet for animal growth, (black) and HC diet (blue). N=8-14 per group, data are represented as mean ± S.E.M., with ** $p<0.01$, *** $p<0.001$, by one-way ANOVA.

Figure S3. A. Gating strategy for the flow cytometric analysis of the CNS during EAE. **B.** Numbers of CNS-infiltrating $CD4^+$ T cell, $CD8^+$ T cell, $ROR\gamma t^+CD4^+$ T cell, activated ($F4/80^+MHC\text{-}II^+$) macrophage and activated ($MHC\text{-}II^+$) microglia from HP, HC, and HF mice. N=10, data are represented as mean ± S.E.M., with * $p<0.05$, ** $p<0.01$, *** $p<0.001$, **** $p<0.0001$, by one-way ANOVA.

Figure S4. Gating strategy for the flow cytometric analysis of splenic T cell cytokine production during EAE.

Figure S5. A. Gating strategy for the flow cytometric analysis of draining lymph node (dLN) during EAE. **B.** Numbers of dLN $CD4^+$ and $CD8^+$ T cells from HP, HC, and HF mice. N=6, data are represented as mean ± S.E.M., with * $p<0.05$, ** $p<0.01$, *** $p<0.001$, **** $p<0.0001$, by one-way ANOVA.

Figure S6. A. Gating strategy for the flow cytometric analysis of dLN *in vitro* re-stimulation experiment. **B-E.** Proliferation of dLN $CD4^+$ (**B**) and $CD8^+$ (**C**) T cells upon *in vitro* re-stimulation quantified by the proportions of cells expressing Ki67 and their corresponding proliferation indices (**D.** $CD4^+$, **E.** $CD8^+$ T cells), with the dashed line denoting proliferation

index of 1. N=6, data are represented as mean ± S.E.M., with * $p<0.05$, ** $p<0.01$, by two-way ANOVA for **B-C**, and one-way ANOVA for **D-E**.

Figure S7. A. Violin plot of the gene set scores for inflammation for CNS-infiltrating immune cells in Ctrl (cyan) and EAE (red) mice analyzed by scRNA-seq. **B.** Violin plots of the gene set scores for inflammation of T cells in PBMCs from Ctrl (pink) and MS patients (blue) analyzed by scRNA-seq.

Figure S8. A. Gating strategy for the flow cytometric analysis of peripheral blood mononuclear cells (PBMCs). **B-C.** Time course analysis of the proportions of BODIPY$^+$ CD4$^+$ (**B**) and CD8$^+$ (**C**) T cells from HP, HC and HF mice quantified by BODIPY^{TM} 493/503 staining. N=8, data are presented as mean ± S.E.M., with **** $p<0.0001$, by two-way ANOVA.

Figure S9. A-B. Correlation analysis for IFNγ (**A**) and IL-17 (**B**) production of dLN CD4$^+$ T cells versus their fat storage quantified by BODIPY^{TM} 493/503 staining. **C.** Correlations analysis for IFNγ production of dLN CD8$^+$ T cells versus their fat storage quantified by BODIPY^{TM} 493/503 staining.

Figure S10. Activation status of conventional dendritic cell (cDC) from mice fed on HP, HC and HF diets quantified by their MHC-II expression level under basal condition (left) and during EAE (right). N=8, data are presented as mean ± S.E.M., * $p<0.05$, *** $p<0.001$, **** $p<0.0001$, by one-way ANOVA.

Figure S11. Violin plots for the expression of *Ppia* in naïve CD4$^+$ T cells from mice fed on diets high in protein (HP), carbohydrate (HC) and fat (HF) analyzed by RNA-seq.

References

1 Senior, A. M., Nakagawa, S., Raubenheimer, D. & Simpson, S. J. Global associations between macronutrient supply and age-specific mortality. *Proc Natl Acad Sci U S A* **117**, 30824-30835, doi:10.1073/pnas.2015058117 (2020).

2 Wali, J. A. *et al.* Determining the metabolic effects of dietary fat, sugars and fat-sugar interaction using nutritional geometry in a dietary challenge study with male mice. *Nat Commun* **14**, 4409, doi:10.1038/s41467-023-40039-w (2023).

3 Ni, D. *et al.* Global associations of macronutrient supply and asthma disease burden. *medRxiv*, doi:10.1101/2023.12.18.23300109 (2023).

4 Wood, S. N. *Generalized Additive Models: An Introduction with R*. (Chapman and Hall/CRC, 2017).

5 Wood, S. N. Fast stable restricted maximum likelihood and marginal likelihood estimation of semiparametric generalized linear models. *J Roy Stat Soc B* **73**, 3-36, doi:10.1111/j.1467-9868.2010.00749.x (2011).

6 Jutta Bolt, R. I., Herman de Jong, and Jan Luiten van Zanden. Vol. GD-174 (Groningen Growth and Development Center, GGDC Research Memorandum, 2018).

7 Diederich, A. Generalized additive models.: An introduction with. *J Math Psychol* **51**, 339-339 (2007).

8 Wood, S. N., Goude, Y. & Shaw, S. Generalized additive models for large data sets. *J Roy Stat Soc C* **64**, 139-155, doi:10.1111/rssc.12068 (2015).

9 Akaike, H. in *Second International Symposium on Information Theory* (Budapest, 1973).
